# The molecular impact of cigarette smoking resembles aging across tissues

**DOI:** 10.1186/s13073-025-01485-x

**Published:** 2025-06-02

**Authors:** Jose Miguel Ramirez, Rogério Ribeiro, Oleksandra Soldatkina, Athos Moraes, Raquel García-Pérez, Winona Oliveros, Pedro G. Ferreira, Marta Melé

**Affiliations:** 1 Department of Life Sciences, Barcelona Supercomputing Center (BSC), C/Jordi Girona 31, Barcelona, 08034 Spain; 2 Universitat de Barcelona, Barcelona, 08034 Spain; 3 Department of Computer Science, Faculty of Sciences, University of Porto, Rua Do Campo Alegre, Porto, 4169-007 Portugal; 4 Laboratory of Artificial Intelligence and Decision Support, INESC TEC, Rua Dr. Roberto Frias, Porto, 4200-465 Portugal

**Keywords:** Cigarette smoking, Gene expression, DNA methylation, Histology images, Aging, Multi-omics, Multi-tissue

## Abstract

**Background:**

Tobacco smoke is the main cause of preventable mortality worldwide. Smoking increases the risk of developing many diseases and has been proposed as an aging accelerator. Yet, the molecular mechanisms driving smoking-related health decline and aging acceleration in most tissues remain unexplored.

**Methods:**

Here, we use data from the Genotype-Tissue Expression Project (GTEx) to perform a characterization of the effect of cigarette smoking across human tissues. We perform a multi-tissue analysis across 46 human tissues. Our multi-omics characterization includes analysis of gene expression, alternative splicing, DNA methylation, and histological alterations. We further analyze ex-smoker samples to assess the reversibility of these molecular alterations upon smoking cessation.

**Results:**

We show that smoking impacts tissue architecture and triggers systemic inflammation. We find that in many tissues, the effects of smoking significantly overlap those of aging. Specifically, both age and smoking upregulate inflammatory genes and drive hypomethylation at enhancers (odds ratio (OR) = 2). In addition, we observe widespread smoking-driven hypermethylation at target regions of the Polycomb repressive complex (OR = 2), which is a well-known aging effect. Smoking-induced epigenetic changes overlap causal aging CpGs, suggesting that these methylation changes may directly mediate the aging acceleration observed in smokers. Finally, we find that smoking effects that are shared with aging are more persistent over time.

**Conclusion:**

Overall, our multi-tissue and multi-omic analysis of the effects of cigarette smoking provides an extensive characterization of the impact of tobacco smoke across tissues and unravels the molecular mechanisms driving smoking-induced tissue homeostasis decline and aging acceleration.

**Supplementary Information:**

The online version contains supplementary material available at 10.1186/s13073-025-01485-x.

## Background

Tobacco smoking causes 8 million deaths annually, representing the primary cause of preventable mortality worldwide [1]. Despite efforts to reduce smoking prevalence, the total number of smokers continues to increase, surpassing 1 billion regular smokers worldwide [1]. Higher mortality in smokers is driven by a threefold increased risk of disease, including respiratory, cardiovascular, metabolic, autoimmune, renal, infectious diseases, and several cancer types [2, 3]. Given its association with age-related diseases, smoking has been suggested to be an aging accelerator [4, 5]. Indeed, cigarette smoking has been associated with premature skin aging [6], accelerated lung function decline [7], telomere attrition [8], oxidative stress [9], and inflammation [9]. Importantly, the increased disease risk does not completely reverse after smoking cessation [10] as 15% of deaths attributable to tobacco smoking occur in ex-smokers [1]. In fact, recent work has shown that smoking triggers enduring alterations in the immune system, particularly with effects in adaptive responses persisting years after cessation [11].

Previous transcriptome and epigenome-wide studies addressing the molecular effects of smoking and the interplay with aging have been restricted to few tissues, mostly airways and whole blood. Transcriptomic studies have identified expression changes associated with both smoking and aging in human airways [12] and murine lungs [13]. DNA methylation clocks that are used to measure the accumulation of DNA methylation changes with age have shown that smoking might accelerate epigenetic aging in lung and whole blood [14–16]. Nevertheless, cigarette smoking effects are not restricted to lung or whole blood [17, 18]. Many tissues, including both directly and indirectly exposed tissues to smoke, have higher numbers of DNA adducts [19] and DNA mutations [20] as well as markers of oxidative damage and inflammation [9]. In addition, smoking-associated DNA methylation changes in lung, colon, and adipose tissues have been shown to mediate gene expression changes that could impact lung function and metabolic health [17, 18]. Finally, smoking-associated diseases involve the cardiovascular, metabolic, and immune systems [3], providing further evidence that the impact of cigarette smoking is systemic. However, what molecular and histological consequences of smoking could lead to aging acceleration in human tissues remains unexplored.

Here, we systematically analyze the transcriptomic, epigenetic, and histological impact of cigarette smoking across human tissues. We use data from the Genotype-Tissue Expression (GTEx) project [21], a resource with RNA-seq data, histology images, and smoking annotation in 46 human tissues across 717 individuals, and DNA methylation arrays from 9 of these tissues. We identify smoking-induced differences in gene expression, alternative splicing, DNA methylation, and tissue architecture across tissues and identify those effects that are simultaneously associated with both smoking and aging. Using ex-smoker information, we classify smoking-associated changes as reversible or non-reversible and address whether smoking effects overlapping those of aging are more persistent. Overall, our multi-tissue and multi-omic analysis provides a comprehensive overview of the systemic and tissue-specific impact of cigarette smoking and how these may drive accelerated tissue aging. Our study sheds light on the long-term health effects and associated risks of smoking, as well as the potential health benefits of smoking cessation.

## Methods

### Data collection

**Fig. 1:**
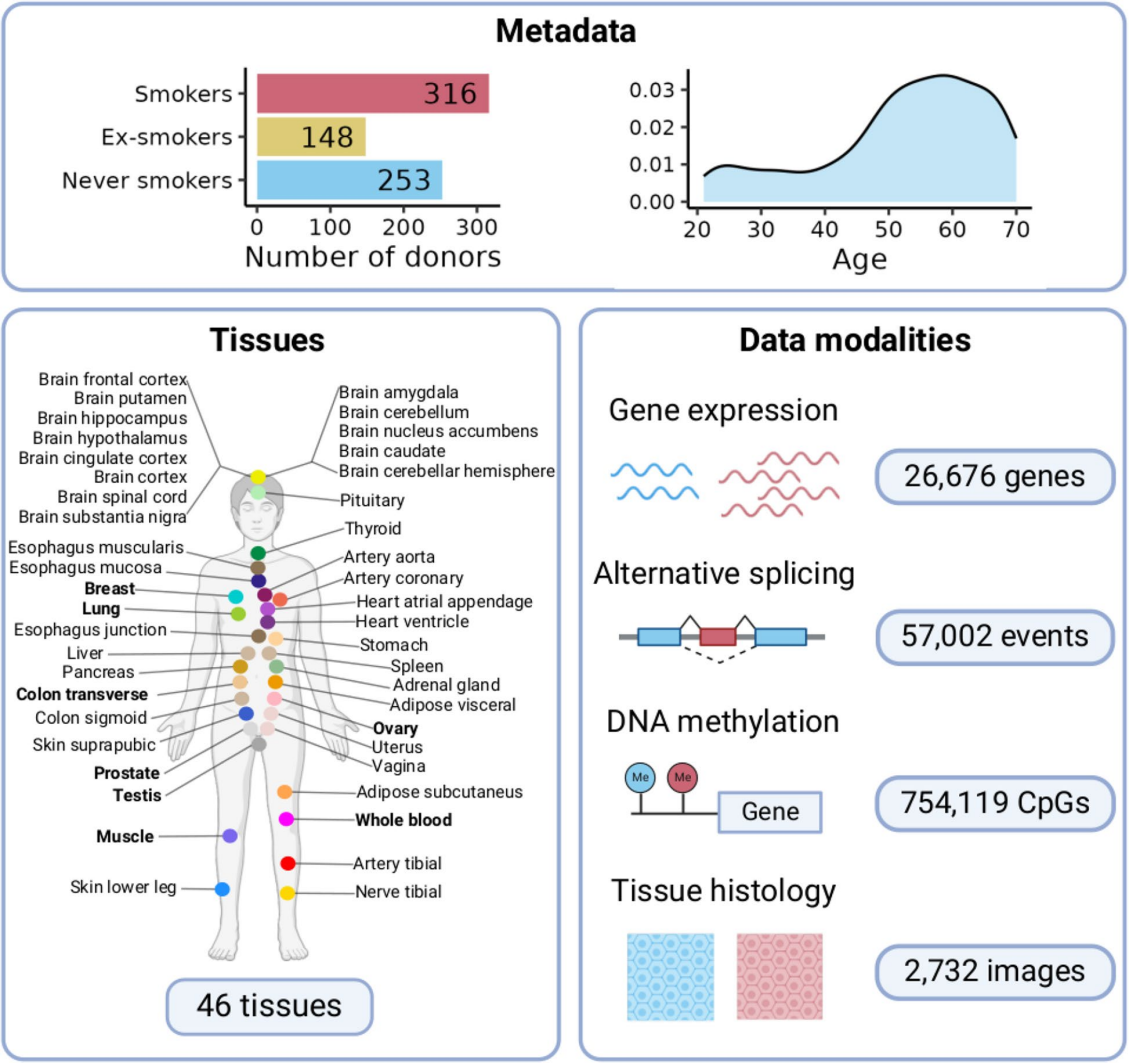
Individuals, tissues, and data modalities analyzed. For all tissues, RNA sequencing and histological images are available. Tissues highlighted in bold also include DNA methylation profiles. We classified GTEx individuals according to their smoking status

We used the Genotype-Tissue Expression (GTEx) v8 data release [21] (Fig. 1, Additional file 1: Table S1). We selected 46 tissues with at least 80 samples comprising 12,654 RNA-sequencing samples from 717 donors, including 316 smokers, 253 never smokers, and 148 ex-smokers (Fig. 1). All human donors were deceased, with informed consent obtained via next-of-kin consent for the collection and banking of deidentified tissue samples for scientific research. The research protocol was reviewed by Chesapeake Research Review Inc., Roswell Park Cancer Institute’s Office of Research Subject Protection, and the institutional review board of the University of Pennsylvania.

### Smoking annotation

The smoking annotation was part of the GTEx v8 protected data stored in dbGap (accession number phs000424.v8.p2) and was obtained either from the donors’ medical record or information provided by the donors’ next-of-kin [22]. We manually curated the annotation and classified donors into never smokers, ex-smokers, and smokers. To do so, we used the original MHSMKSTS variable, which encoded information on whether the donor ever smoked. We excluded 25 donors with unknown smoking status. Only cigarette-smoking donors were considered according to the variable MHSMKTP, so we excluded 22 donors annotated as smokers of “Cigar,” “Pipe,” or “Other.” We revised the manual comments under MHSMKCMT to distinguish individuals that quit smoking, which we reannotated as ex-smokers. The variable MHSMKCMT is free text annotated by the donors’ next-of-kin, where the maximum time between smoking cessation and death is more than 30 years, and the minimum is 1 month. From the MHSMKCMT annotations, we further excluded 43 donors with unclear annotations. These excluded annotations include more smokers of non-cigarettes, individuals that smoked for a very short period of time in the past, and intermittent smokers. We restricted the analysis to tissues with at least 80 samples with annotation for all the variables included in our models. In total, we analyzed 11,962 RNA-seq samples from 46 human tissues and 717 post-mortem donors. From the 717 donors, 316 are smokers that smoked a median of 20 cigarettes per day (age mean = 49.9, sd = 13.3), 148 ex-smokers (age mean = 56.9, sd = 11.7), and 253 never smokers (age mean = 52.6, sd = 12.7).

### Gene and alternative splicing event quantification

Gene and transcript quantifications were based on the GENCODE 26 release annotation (https://www.gencodegenes.org/human/release_26.html) [23]. We downloaded gene counts and TPM quantifications from the GTEx portal (https://gtexportal.org/home/downloads/adult-gtex) [24]. We selected genes with the protein-coding and lincRNA biotype on the GTEx GENCODE v26 gtf file. For the expression analysis, we considered expressed genes per tissue (TPM ≥ 0.1 and ≥ 6 reads in ≥ 20% of tissue samples), excluding genes in the pseudoautosomal region (PAR). In total, we analyzed 26,676 genes (19,255 protein-coding and 7421 lincRNA) across tissues. For the splicing analysis, we downloaded transcripts TPM quantifications from the GTEx portal (https://gtexportal.org/home/downloads/adult-gtex) [24] and we used SUPPA2 [25] to calculate percentages of splicing inclusion (PSI) for 7 different types of splicing events: skipped exon, mutually exclusive exons, alternative 3 prime, alternative 5 prime, retained intron, alternative first exon, and alternative last exon. Specifically, we used SUPPA2 to first generate the dictionary of splicing events from the GENCODE v26 annotation and then computed their PSI values for each sample and splicing event. Each splicing event is defined by a set of isoforms: those that include the exonic/intronic sequence (spliced-in isoform) and those that either exclude it or include an alternative exonic sequence (spliced-out isoform). We used the following criteria to select the alternatively spliced events (ASEs) in each tissue: events in protein-coding and lincRNA genes expressed in each tissue; events quantified in all tissue samples; we excluded events with low complexity (fewer than 15 PSI unique values) or insufficient variability (the most common event is 4 times more common than the second most common); we kept events from expressed isoforms (TPM ≥ 0.5 in ≥ 20% of tissue samples for both the most abundant spliced-in and spliced-out isoforms) and with a quantifiable contribution of the traits of interest (see *Hierarchical partition analysis*). In total, we tested 57,002 alternative splicing events (16,704 skipping exons, 1142 mutually exclusive exons, 6491 alternative 5′ splice sites, 7412 alternative 3′ splice sites, 3919 intron retentions, 16,681 alternative first exons, and 4653 alternative last exons) across tissues.

### Differential expression analysis

To identify differentially expressed genes (DEGs) we used linear-regression models following the *voom-limma* pipeline [26, 27]. We corrected for technical covariates routinely included in previous GTEx publications [28, 29]. These covariates are related to parameters of donor death (Hardy scale), ischemic time, RNA integrity number (RIN), and sequencing quality control metrics (Exonic rate). We further controlled for unknown sources of variation, mainly related to differences in tissue composition and sequencing batch, by including the first two PEER factors, as PEER1 mostly correlates with cell type heterogeneity (see Fig. S4 A from [30]) and PEER2 with sequencing batch (see Fig. S9a from [31]). Furthermore, conversely to eQTL discovery, the effect of including additional PEER factors on the DEGs discovery is variable across tissues and leads to reduced power to detect expression differences [32]. We also included in the model four demographic traits: the donors’ genetically inferred ancestry [21] (either European American, African American or admixed donors), sex (either male or female), age, and body mass index (BMI). Ancestry and sex were treated as categorical variables, whereas age and BMI were treated as continuous variables.

We also corrected for clinical traits obtained from histopathological annotations, publicly accessible from the GTEx portal [33, 33]. However, we only included in the final models the clinical traits with more than 20 diseased individuals associated with changes in gene expression in a simpler model. We first modeled each clinical trait per tissue, correcting by the previously mentioned covariates, and kept the ones that retrieved at least 5 DEGs (Additional file 2: Table S2). Finally, we ran one model per tissue, correcting by all the technical covariates, the four demographic traits, the chosen clinical traits, and smoking.

We compared log-cpm gene expression values and evaluated the statistical significance of smoking and the four demographic traits. Smoking was defined as a categorical variable with three levels: never smokers, ex-smokers, and smokers, and all possible contrasts were evaluated. We corrected all analyses for multiple testing using the false discovery rate (FDR) through the Benjamini–Hochberg method and considered genes differentially expressed at an adjusted p-value below 0.05.

$$log\mathit2\left(cpm\right)\sim HardyScale+IschemicTime+RIN+ExonicRate+PEER1+Peer2+Clinical\;traits+Ancestry+Sex+Age+BMI+Smoking$$

When downsampling, we used the same models using an equal number of never smokers and smokers in sets of 50 different permutations. We first performed 50 iterations, downsampling to 36 never smokers and 36 smokers, which is the minimum number needed to evaluate all tissues. We then downsampled to 50, 80, and 100 samples per category. To report the results, we computed the mean across the 50 permutations.

To validate the genes differentially expressed between smokers and never-smokers, we compared our results to other studies that used independent transcriptome datasets. To obtain a list of previously reported smoking-DEGs in lung, we downloaded and parsed Table S1 from Bosse et al. [34], Supplementary Table S3 A and S3B from Landi et al. [35], and Table S1 from Pintarelli et al. [36]. For blood, we downloaded and parsed Table S2 from Huan et al. [37] and Table S2 from Vink et al. [38]. For adipose tissue, we downloaded and parsed Table 2 from Tsai et al. [17]. To assess the replicability of our results, we identified smoking-DEGs with GTEx data using similar filtering criteria (logFC and FDR thresholds) as those used in each paper. We performed a two-tailed Fisher’s exact test to test if the number of DEGs we identified significantly overlapped with the genes identified in previous studies. A gene was only considered overlapping if it was DE in the same direction in both studies. We used the protein-coding and lincRNA genes expressed in the respective tissue as background.

### Additive and interaction effects

In order to investigate additive effects, we conducted one-tailed Fisher’s exact tests on the common DEGs with smoking and each demographic trait per tissue. We used as background the genes expressed in each tissue (TPM ≥ 0.1 and ≥ 6 reads in ≥ 20% of tissue samples). We ran the analysis per tissue and for each pair combination of the smoking variable and a demographic variable. The cross-tabulations are as follows using age as the demographic trait of example:

**Table:** 

	Age-DEGs	Non age-DEGs
Smoking-DEGs	*X*	*Z*
Non smoking-DEGs	*Y*	Background − *X* − *Y* − *Z*

where *X* would correspond to the number of smoking-age-DEGs, *Y* would correspond to the number of age DEGs minus the number of smoking-age-DEGs, and *Z* would correspond to the number of smoking DEGs minus the number of smoking-age-DEGs. We derived the odds ratio (OR) from these tables computed as follows:

$$\mathrm O\mathrm R\;=\frac{Y\times Z}{X\times(\mathrm{Background}-X-Y-Z)}$$

The interpretation of the odds ratio is as follows: if the OR is higher than 1, age-DEGs and smoking-DEGs overlap more often than expected if the two variables were independent. If the OR is lower than 1, the two variables overlap less than expected by chance.

The *p*-values were corrected using the Benjamini–Hochberg method, correcting for all the tests performed across tissues per demographic trait. We considered significant effects with a false discovery rate (FDR) below 0.05. To then check if the direction of change was concordant between smoking and the demographic traits, we conducted a chi-square test and corrected for multiple testing in the same way. In this case, from the subset of smoking-age-DEGs, the contingency table would be as follows:

**Table:** 

		Age-DEGs	
		Upregulated	Downregulated
Smoking-DEGs	Upregulated	*X*	*Z*
	Downregulated	*Y*	*B*

To investigate interactions, we expanded the linear models described in the section “ Differential expression analysis” in each tissue, adding interaction terms between smoking and the other demographic traits (Ancestry:Smoking + Sex:Smoking + Age:Smoking + BMI:Smoking), for the smoking contrast that compares never smokers and smokers. For an interaction to be tested in a tissue, we required (1) previously found DEGs with both traits involved in the interaction term, and (2) sufficient sample size, which we define as at least 20 samples per group. To determine the number of samples in each combination, we categorized the continuous variables. Age was split into two groups: young (age < 45) and old (age ≤ 45). BMI was divided into three groups: normal (BMI < 25), overweight (25 ≤ BMI < 30), and obese (BMI ≥ 30).

In order to identify the interaction effects between smoking status and genotype on gene expression, we followed the steps in [39]. For each of the gene-variant pairs identified as independent cis-eQTLs in GTEx v8 [21], we ran a linear regression model including genotype, smoking status, and covariates to test the effect of the interaction term Genotype × Smoking on gene expression separately in each tissue. We tested only the gene-variant pairs that had at least 3 samples in every combination. Each tissue was corrected for a different number of PEER factors based on the tissue sample size, as described in the GTEx eQTL analysis [21]. For implementation, we used tensorQTL [40], where we could model separately for each gene and account for multiple testing errors using Benjamini–Hochberg correction with FDR < 0.05.

### Differential splicing analysis

To perform differential splicing analysis, we used a similar method to the one described in [32] to allow both a direct comparison with the differential gene expression analysis and a subsequent quantification of the alternative splicing variation explained by each trait (see *Hierarchical partition analysis*). In short, we modeled percentage of spliced-in (PSI) values with fractional regressions. This method is suited to work with bounded values that can assume the extremes, as is the case for PSI values in 0 and 1. Specifically, we used the R *glm* function from the R package *stats * [41] setting *family* = *‘quasibinomial* (“logit'’)’ as a parameter. For each splicing event within each tissue, we fitted logit transformed PSI values with the same covariates used in differential expression analysis and evaluated the statistical significance of smoking and the demographic traits of interest: ancestry, sex, age, and BMI.

$$PSI\;values\sim HardyScale+IschemicTime+RIN+ExonicRate+PEERI+PEER2+Ancestry+Sex+Age+BMI+Smoking$$

To calculate robust standard errors for our coefficients, we used the *vcovHC* function from the R package *sandwich * [42] with *type* = *"HC0"*. To do multiple comparisons for the variables with three levels (e.g., to compute the DSEs between never smokers and smokers, between ex-smokers and smokers, and between never smokers and ex-smokers), we used the function *glht* from the package *multcomp * [43], with Tukey’s all-pair comparisons for the *mcp* function. We corrected all analyses for multiple testing using false discovery rate (FDR) through the Benjamini–Hochberg method implemented in the R package stats [41]. We considered events differentially spliced at an adjusted *p*-value (FDR) below 0.05.

To investigate the functional consequences of DSEs, we first identified the isoforms that contribute to each splicing event. From these sets of isoforms, we selected the most abundant isoforms per tissue that include (spliced-in) and exclude (spliced-out) the splicing event. Depending on the biotype of these two isoforms, DSEs can then be associated with a switch between protein-coding isoforms, a switch between a protein-coding and a non-coding isoform, or a switch between non-coding isoforms. For those events with a switch between protein-coding isoforms, we executed the pipeline from [32] to analyze the functional consequences of the switches based on functional domains from Pfam [44]. The percentage of events that belong to each switch type was computed using the sum of the events across tissues from the given switch type over the total number of events, without considering repeated events present due to tissue sharing. To test if smoking tends to be associated with an increase in non-coding isoforms, we used a binomial test with a probability of 0.5 as the null hypothesis.

### Functional enrichment

We used the R package *clusterProfiler * [45] for the different overrepresentation enrichment analyses (ORA) conducted throughout the paper. For each ORA, we defined as background all the expressed genes in the given tissue and analyzed upregulated and downregulated genes independently. We corrected for multiple testing by using the Benjamini–Hochberg method and considered as significant gene sets with an FDR < 0.05. We performed this analysis across several ontologies, including gene ontology (GO), KEGG, and disease ontology (DO). To summarize the enriched GO terms, we employed the *orsum* package for up- and down-regulated terms separately [46]. The analysis was performed by considering all the enriched terms of each tissue at the same time, allowing for an easier comparison between enrichment results.

### Machine learning on histological images

We used the histological images of lung, thyroid, pancreas, and esophagus mucosa available in the GTEx Histological Image Viewer (https://gtexportal.org/home/histologyPage) [33], with the aim of building tissue-specific classifiers of smokers and never smokers. For each donor, we downloaded the respective whole slide images (WSI) and divided them into tiles of size 512 × 512 × 3 using *PyHist* [47] with 2 × for the downsample parameter. We kept the tiles with at least 85% tissue content.

We divided the dataset into train and test with the test set being composed of a subset of donors (n = 85) common between the 4 tissues. This step ensures a more robust model comparison. In each tissue, we used 80% of the train subjects for model training, while we kept the remaining 20% for model validation. For modeling we used the pre-trained convolutional neural network (CNN) Xception [48] changing only the top layers by adding a Max Pooling [49], a Dropout [49], and a sigmoid activation [49] to get the probability of each subject being a smoker. All models were compiled utilizing a weighted binary cross entropy as loss function, and an *Adam* algorithm with learning rate of $1\times {10}^{-4}$ as an optimizer for the gradient descent. For each tissue we adjusted different class weights in order to mitigate the impact generated by the imbalanced distribution of cases in the final results (see GitHub). The best model for each tissue was selected based on AUC.

### Thyroid follicle detection

We developed a custom CellProfiler [50] pipeline to detect and measure thyroid follicles. The first step consists of the computational application of the Ponceau-Fuchsin (PF) stain to the original Hematoxylin and Eosin (H&E) stained image tiles. The PF stain hue absorption helps isolate colloid-containing follicles when using adaptive thresholding in grayscale. We further manually adjusted IdentifyObject module parameters in order to identify the separate follicles. On identified follicles, we used the *ObjectMeasurement* module to calculate area, shape, and size statistics on all thyroid tiles.

### Cell-type deconvolution

In order to perform cell-type deconvolution of the GTEx bulk dataset, we first obtained the processed single-cell dataset from GEO GSE173896 [51]. This dataset integrated 12 individuals (6 smokers, 3 never smokers and 3 ex-smokers) and > 57,000 cells classified into 30 discrete cell types.

Before deconvolution, we processed the single-cell dataset by removing rare cell types (cell types with less than 50 cells across the dataset), and only protein-coding and lincRNA genes in common with the bulk dataset were kept. Next, we used the *cleanup.genes* function from the *BayesPrism* R package [52] to further exclude ribosomal, mitochondrial, and sex-chromosome genes, as per package instructions, as well as genes expressed in less than 50 cells. We merged subcell types into unified cellular populations, and identified marker genes using the *get.exp.stat* function. Marker genes with a log fold change > 0.1 and *p*-values < 0.01 were selected. Finally, cellular deconvolution was performed based on the expression of these genes.

To compare the macrophage populations between smokers’ groups, while controlling for other sources of variation, we fitted a beta regression model on the macrophage proportions in each individual:

$$CelltypeProportion\sim HardyScale+IschemicTime+Ancestry+Sex+Age+BMI+atelectasis+emphysema+fibrosis+pneumonia+Smoking$$

We evaluated the statistical significance of smoking using the *glht* function from the package *multcomp * [53]. We considered the difference significant at a nominal *p*-value < 0.05.

### Differential methylation analysis

We downloaded normalized beta counts of the 754,054 CpGs from the Infinium MethylationEPIC array generated in [54]. We correlated smoking status with their provided PEERs and found significant correlations with PEER3 and PEER4 (Additional file 3: Fig. S4a). Hence, we only corrected for PEER1 and PEER2. We used *limma * [27] to run linear models on *M* values [55] and corrected for the following set of covariates to be as similar as possible to the previous analysis:

$$M\;values\sim HardyScale+Ischemic+PEER\mathit1\mathit+PEER\mathit2\mathit+Clinical\mathit\;traits\mathit+Ancestry\mathit+Sex\mathit+Age\mathit+BMI\mathit+Smoking$$

To perform functional enrichment with the EPIC array, we need to take into account that some genes contain more probes than others. For this goal, we used the function *gometh* from the *missMethyl* package [56] to get GO:BP terms using as background all 754,054 positions studied from the array.

The overlap between smoking and the other demographic traits was followed in the same way as in the “ Differential expression analysis” section. We also tested interactions but did not find any significant results**.**

To validate the DMPs between smokers and never smokers, we compared our results to those obtained with independent datasets using arrays. For lung, we used the results in Table 1 obtained using the 450 K array from [57], we downloaded the EPIC array data available in Table S2 from [58], and the 450 K cancer results in Table S1 from [59]. For blood, we used the data on the 450 K from [60] available in Table S2, the data on EPIC in Table S1 from [61], and the data on http://mimeth.pasteur.fr/ from [62]. For the adipose tissue, we downloaded Table S1 from [17] on the 450 K array. We performed two-tailed Fisher’s exact tests to check if the DMPs we identified overlapped with the positions observed in previous studies, with the same direction of effect. The background was either the set of studied positions from the EPIC array or the subset of positions available in the EPIC array that were also probed in the 450 K array.

We tried to identify interaction effects between smoking status and genotype on DNA methylation following similar steps to the “ Differential expression analysis” section, but we did not find significant results after Benjamini–Hochberg correction with FDR < 0.05*.*

### Annotation of DMPs

The DMPs were classified depending on their location at promoters, enhancers, gene bodies, or intergenic, based on the annotations provided in the EPIC v1.0 array manifest b5. Positions annotated as “Promoter_Associated” under “Regulatory_Feature_Group” or under “TSS200” or “TSS1500” under “UCSC_RefGene_group” were assigned as promoters. From the rest, the ones with any value in “Phantom5_Enhancers” were assigned as enhancers. From the rest, the positions including “Body,” “1 stExon,” “ExonBnd,” “3UTR,” or “5UTR” under “UCSC_RefGene_group” were annotated as gene bodies. The rest were annotated as intergenic. We also classified the DMPs into CpG islands, CpG shore, CpG shelf, or open sea, using the variable “Relation_to_UCSC_CpG_Island” from the EPIC manifest. To study the enrichment of smoking-DMPs or smoking-age-DMPs in the different genomic locations, we performed Fisher’s exact test for each region separately for hypomethylated and hypermethylated DMPs and adjusted for multiple testing using Benjamini–Hochberg correction.

To annotate the chromatin states around each array probe, we used the 18 chromatin states inferred with ChromHMM [63, 64] for the lung sample BSS01190 generated by the ROADMAP Epigenomics consortium [63] and analyzed for EpiMap [65]. TssFlnkD, TssFlnk, and TssFlnkU are reported together under “Flanking TSS,” EnhA1 and EnhA2 under “Active enhancer,” and EnhG1 and EnhG2 under “Genic enhancer.” For the blood analysis, we used the PBMC sample BSS01419.

The data was downloaded from https://personal.broadinstitute.org/cboix/epimap/ChromHMM/observed_aux_18_hg19/CALLS/ [66]. To perform enrichment of DMPs in the different chromatin states, we performed Fisher’s exact test similarly to the analysis on the different genomic locations.

To study the enrichment of transcription factor binding sites around DMPs, we downloaded the processed CHIP-seq data on transcription factors for the human v19 lung available in ChipAtlas [67]. We performed Fisher’s exact test for each transcription factor separately for hypomethylated and hypermethylated DMPs, and adjusted for multiple testing using the Benjamini–Hochberg correction. The transcription factors that are part of the Polycomb repressive complex available in the CHIP-seq data were EZH12, SUZ12, YY1, KDM2B, REST, PCGF2, CBX2, CBX8, RNF2, and RYBP.

To study the enrichment in causal CpG, we downloaded the causal CpG reported by Ying et al. [68]. For the set of CpG associated with each trait, we computed the significance of the overlap with smoking-CpG and aging-CpG, using Fisher’s exact test. To test the direction of the effect, we classified each causal CpG as protecting or damaging, according to Ying et al., by checking the direction of the product of the Mendelian randomization effect (*b*) and age effect (*B*). Sites with b*B > 0 were defined as protective, whereas sites with b*B < 0 were defined as damaging. We conducted an overlap analysis with the smoking-DMPs and aging-DMPs using the Fisher test. Hyper- and hypomethylated positions were tested independently, and* p*-values were adjusted for multiple testing.

### Correlation between DNA methylation and gene expression

The CpG-gene pairs were retrieved first from the EPIC v1.0 manifest b5. We considered a probe to be part of a pair for every gene annotated under “UCSC_RefGene_Name.” For the probes annotated as a promoter or enhancer that did not have any assigned genes in the manifest, we annotated the closest gene using the function *matchGenes* from *bumphunter * [69]. Note that we will be using the terminology “CpG-associated gene” for the following definitions: either there is a known previous association reported by Illumina in the EPIC manifest (98% of CpG-gene pairs) or the gene is the closest to a promoter or enhancer (2%). We computed Pearson’s correlation for the DMP-DEG pairs and corrected for multiple testing using the Benjamini–Hochberg method. We considered significant correlations with an FDR < 0.05. To compute the percentage of DEGs that significantly correlate with DMPs, we considered DEGs associated with at least one probe in the array.

### Multi-Omics Factor Analysis (MOFA)

Gene expression and DNA methylation data derived from the EPIC array from the lung tissue of smokers and never smokers were integrated using MOFA [70]. Before the analysis, methylation features were separated in promoters, enhancers, gene body and intergenic features based on the annotations provided in the EPIC v1.0 manifest, as previously described. Each set of methylation features were treated as distinct data modalities. Before data integration, gene expression count data was normalized using variance stabilizing transformation, using the *vst* function from DESeq2 [71] package, while methylation data was transformed into M-values as previously described. For each data modality, we selected the top 5,000 most variable features. Data was scaled prior to model training. We allowed MOFA to select 12 latent factors, with factors explaining less than 0.01 variance being pruned.

After model training, the latent factors were correlated (Spearman rho) with demographic and technical traits (sex, age, BMI, smoking status, and PEER factors). We considered a latent factor significantly correlated with a trait at an FDR < 0.05, after multiple test corrections across all demographic trait–latent factor combinations.

### Enrichment analysis of MOFA factors

MOFA infers a low-dimensional representation of the data by identifying latent factors that capture the primary sources of variability across multiple modalities. This process generates a weight matrix, where each feature (i.e., gene or CpG) is assigned a weight for each latent factor, reflecting its contribution to the underlying factor. We leveraged this weight matrix across data modalities and performed gene set enrichment analysis for GO terms using the *run_enrichement* function from the MOFA package (Argelat et al.). We performed the analysis for positive and negative weights independently. To test for functional enrichment in CpGs, we used their associated gene in the “UCSC_RefGene_Name” column from the EPIC v1.0 manifest b5. We corrected for multiple testing in each analysis and considered terms significant at an FDR < 0.05. Finally, enriched terms across all analyses were summarized using the *orsum* package [46].

### Analysis on differentially methylated regions

We downloaded the WGBS data from dbGaP under phs000424 [72]. We analyzed the tissues with at least 3 never smoker and 3 smoker samples (Additional file 3: Fig. S6c). We trimmed the adapters with Trim Galore with a quality cutoff of 30 [73]. We aligned the reads to hg38 with Bismark [74] using default parameters. We used bismark_methylation_extractor to summarize the number of reads supporting a methylated cytosine and an unmethylated cytosine for every cytosine present in the reference genome, using the parameters “–ignore 2 –ignore_r2 3 –ignore_3prime 1 –ignore_3prime_r2 3” [72]. This summarizes the evidence of methylation at each cytosine in the human reference genome.

We used *bsseq* [75] to call differentially methylated regions between smokers and never smokers following a previously published pipeline [72]. In short, we used *BSmooth* [75] to estimate the CpG methylation levels. We ran a small smooth function to estimate the methylation level in all small genomic regions (smoothing over windows of at least 1 kb containing at least 70 CpGs). Then, we analyzed all CpGs that had a sequencing coverage of at least 1 read in all samples for a total of 16,290,182 CpGs. Finally, we computed t-statistics and identified DMRs by thresholding the *t*-statistics following the *bsseq* pipeline [75]. We only considered the DMRs that contained at least 5 CpGs with an absolute logFC threshold higher than 0.1, following *bsseq* guidelines.

To study the enrichment of Polycomb targets, we used the same approach described under the “Annotation of DMPs” section. For thyroid, we used the chromatin states generated by the ROADMAP Epigenomics consortium [63] and analyzed for EpiMap [65] of sample BSS01832; BSS01124 for hippocampus, BSS00369 for frontal cortex, BSS00173 for caudate, and BSS01124 for amygdala, which corresponds to the hippocampus sample, since there was no exact match of the sampling tissue. To study the enrichment of DMRs in CpG islands, we used *annotatr* [76] to get the annotation of CpG islands in hg38, and we overlapped the DMRs and the CpG island regions with GenomicRanges [77].

### Age prediction with methylation clocks

To determine biological age, we considered the well-established Horvath clock [78], as well as three recently published models: CauseAge, DamAge, and AdaptAge [68]. We obtained the features and coefficients for each model from the supplementary data in the respective publications. Across all models, we found missing features in our dataset. As such, before model prediction, we performed a data imputation step by taking the mean of the across the sample. We considered as background all the probes from the Illumina Infinium HumanMethylation27 for the Horvath models and the Illumina Infinium HumanMethylation450 for the remaining models. This choice was motivated by the technology used to generate the training data in each study. Due to the high percentage of missing features in CauseAge (211/586, ~ 36%) we excluded this model from our analysis. For the Horvath model, we further normalized the dat, following the original author protocol [78].

After the age prediction step, we computed the biological age acceleration as the difference in model error for each sample (predicted age − chronological age) similar to the approach taken by other authors [14, 78]. We then compared the errors between smokers and never smokers, adjusting for potential confounding factors using a linear regression:

$$Error\sim HardyScale+IschemicTime+Ancestry+Sex+Age+BMI+Smoking$$

For each model, we computed the significance of the smoking variable using a *T*-test. We corrected for multiple testing in each biological clock. We determined a result to be significant at FDR < 0.05.

### Classification into different reversibility categories

To assess the reversibility of gene expression, alternative splicing events, and DNA methylation, we compared DEGs, DSEs, and DMPs between the three groups: never smokers, ex-smokers, and smokers. Based on these comparisons, we classified the events into three categories:

- Reversible: smoking-DEGs, DSEs, or DMPs (identified in the never smokers vs. smokers comparison) were also differentially expressed, spliced, or methylated in ex-smokers vs. smokers, with the same directional change, but not in ex-smokers vs. never smokers. This classification ensures that the expression, splicing, or methylation levels in ex-smokers are intermediate between those in never smokers and smokers.
- Non-reversible: Smoking-associated DEGs, DSEs, or DMPs were also differentially expressed, spliced, or methylated in never smokers vs. ex-smokers (in the same direction) but not in ex-smokers vs. smokers, suggesting persistent alterations.
- Partially reversible: Events that did not meet the criteria for either reversible or non-reversible classification.

For partially reversible genes, we further compared the mean absolute logFC obtained in the never smokers vs. ex-smokers analysis with the one obtained in the ex-smokers vs. smokers analysis through a two-tailed Wilcoxon paired test.

### Machine learning on gene expression and methylation data

All the machine learning models based on gene expression and methylation were trained using the gradient boosting algorithm implemented in the *lgb.train* function from the *lightgbm* R package.

For gene expression, values were normalized in log2 scale, log2(TPM + 1) and only protein-coding and lincRNA genes were considered as input. For methylation data, all the probes in lung data were considered. In order to evaluate model performance, a fivefold cross-validation scheme was implemented.

For each tissue, we trained separate models using gene expression or DNA methylation data for each tissue. In instances where the model achieved high accuracy (cross-validated mean AUC > 0.85), we trained a final model using all available samples from smokers and never smokers. Classification of ex-smoker samples into never smokers or smokers was performed using the final model.

## Results

### Cigarette smoking impacts gene expression and alternative splicing in most human tissues

**Fig. 2:**
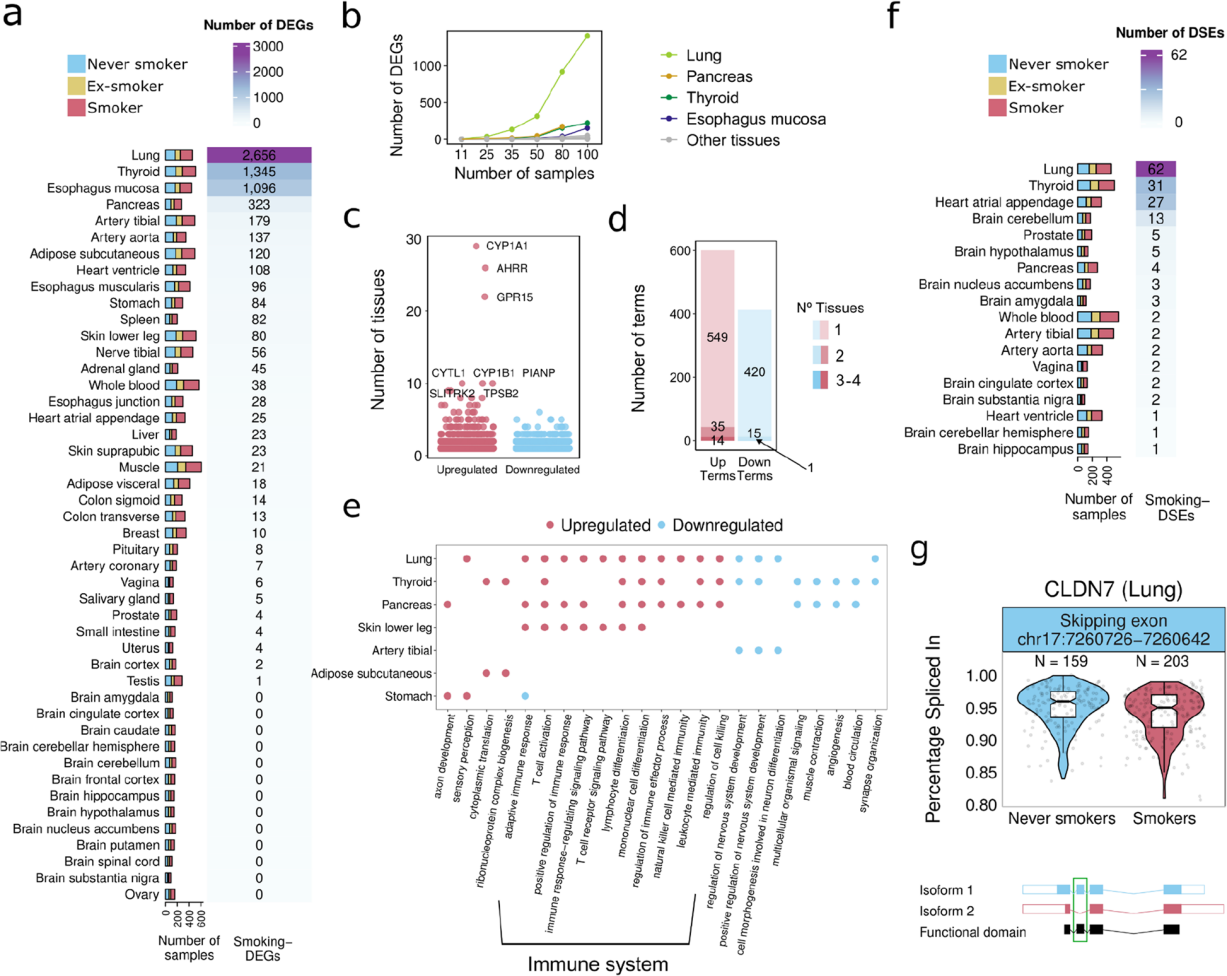
Smoking differential gene expression and alternative splicing analysis. **a** Tissue sample size (left) and number of smoking-DEGs per tissue (right). **b** Number of smoking-DEGs per tissue at varying sample sizes while keeping an equal number of smokers and never smokers. **c** Number of tissues in which the smoking-DEGs are differentially expressed. Gene names are shown for smoking-DEGs in more than 9 tissues. **d** Number of tissue-specific and tissue-shared gene ontology enriched terms (FDR < 0.05, minimum gene count > 5). **e** Most enriched and summarized gene ontology terms (biological processes) for smoking up- (red) and down- (blue) regulated genes. **f** Tissue sample size (left) and number of smoking-DSEs per tissue (right). **g** PSI values in smokers and never-smokers for a skipped exon in *CLDN7* (top) and visual representation of the two most expressed isoforms involved in the exon skipping event (bottom). Isoform 1 is ENST00000360325 and Isoform 2 is ENST00000538261

The transcriptomic impact of cigarette smoking has been studied in a limited number of tissues [12, 38]. To perform a systematic quantification of the effect of cigarette smoking on gene expression across human tissues, we used the Genotype-Tissue Expression (GTEx) v8 data release [21] (Fig. 1, Additional file 1: Table S1). We selected 46 tissues with at least 80 samples comprising 12,654 RNA-sequencing samples from 717 donors, including 316 smokers, 253 never smokers, and 148 ex-smokers (Methods). We performed differential gene expression analysis per tissue, comparing smokers and never smokers and controlling for technical and biological covariates, including cell-type composition (Methods, Additional file 2: Table S2). The number of smoking-associated differentially expressed genes (smoking-DEGs) varied across tissues (FDR < 0.05, Fig. 2a, Additional file 3: Fig. S1a, Additional file 4: Table S3). Lung (2656 smoking-DEGs) followed by thyroid (1345 smoking-DEGs) and esophagus mucosa (1096 smoking-DEGs) had the largest number of smoking-DEGs. Downsampling to an equal number of samples per tissue showed consistent results, with lung, pancreas, thyroid, and esophagus mucosa having the largest number of smoking-DEGs (Fig. 2b, Additional file 3: Fig. S1b). A similar pattern was observed when applying log fold change cutoffs to the differentially expressed genes (Additional file 3: Fig. S1c). Importantly, we were able to reproduce previous findings, as the smoking-DEGs here identified significantly overlap with those identified in previous studies for the same tissue (two-tailed Fisher’s exact test; *p*-value < 0.05) (Additional file 3: Fig. S1 d) [17, 34–38].

Smoking effects are largely tissue-specific, with 86% of smoking-DEGs altered in a single tissue (Fig. 2c). However, 8 genes were found to be upregulated in 9 or more tissues (Fig. 2c, Additional file 3: Fig. S1e). Of these, *AHRR*, *CYP1 A1*, and *CYP1B1* are known to be upregulated upon direct exposure to polycyclic aromatic hydrocarbons (PAHs) [79], suggesting that tobacco toxic compounds reach tissues not directly exposed to tobacco smoke. Other highly shared and upregulated genes, such as *GPR15*, *PIANP*, *CYTL1*, and *TPSB2*, are involved in immune system functions and inflammation [80–83]. Functional enrichment showed little overlap across tissues, indicating that most smoking-altered pathways are tissue-specific (Fig. 2d, Additional file 5: Table S4, Additional file 6: Table S5). For example, genes downregulated in the lung are associated with cilium assembly pathways, consistent with reported cilia dysfunction in smokers [84], and downregulated genes in the thyroid are associated with cancer-related pathways, consistent with smokers being at lower risk of developing thyroid cancer [85] (Fig. 2e, Additional file 3: Fig. S2). The few pathways that are shared across tissues are mostly for upregulated genes and associated with immune function, consistent with increased systemic inflammation in smokers [86–88] (Fig. 2e).

Alternative splicing has been associated with tobacco smoking in blood [89, 90]. We computed the “percentage spliced-in” (PSI) for seven types of alternative splicing events [25] and performed differential analysis to test the association between PSI variation and smoking, while correcting for known sources of technical variation (Methods). We found differentially spliced events (smoking-DSE) in 17 tissues (Fig. 2f, Additional file 7: Table S6). The most affected tissues were lung (65 smoking-DSE), thyroid (34 smoking-DSE), and heart (27 smoking-DSE). In half (48%) of the smoking-DSEs, both the inclusion and exclusion of the event are associated with at least one protein-coding isoform, of which 46% are associated with direct changes in annotated protein domains [44] (Methods). One example is *CLDN7*, a gene associated with various cancer types, including lung cancer [91]. Most of the other half of smoking-DSEs (44%) are changes between protein-coding and non-coding isoforms, with smoking being associated with the loss of coding potential (64%, binomial test; *p*-value = 0.015).

Overall, these results show that smoking affects gene expression and alternative splicing in multiple tissues, leading to both tissue-specific changes and systemic inflammation.

### Cigarette smoking induces histological changes in the lung and thyroid

**Fig. 3:**
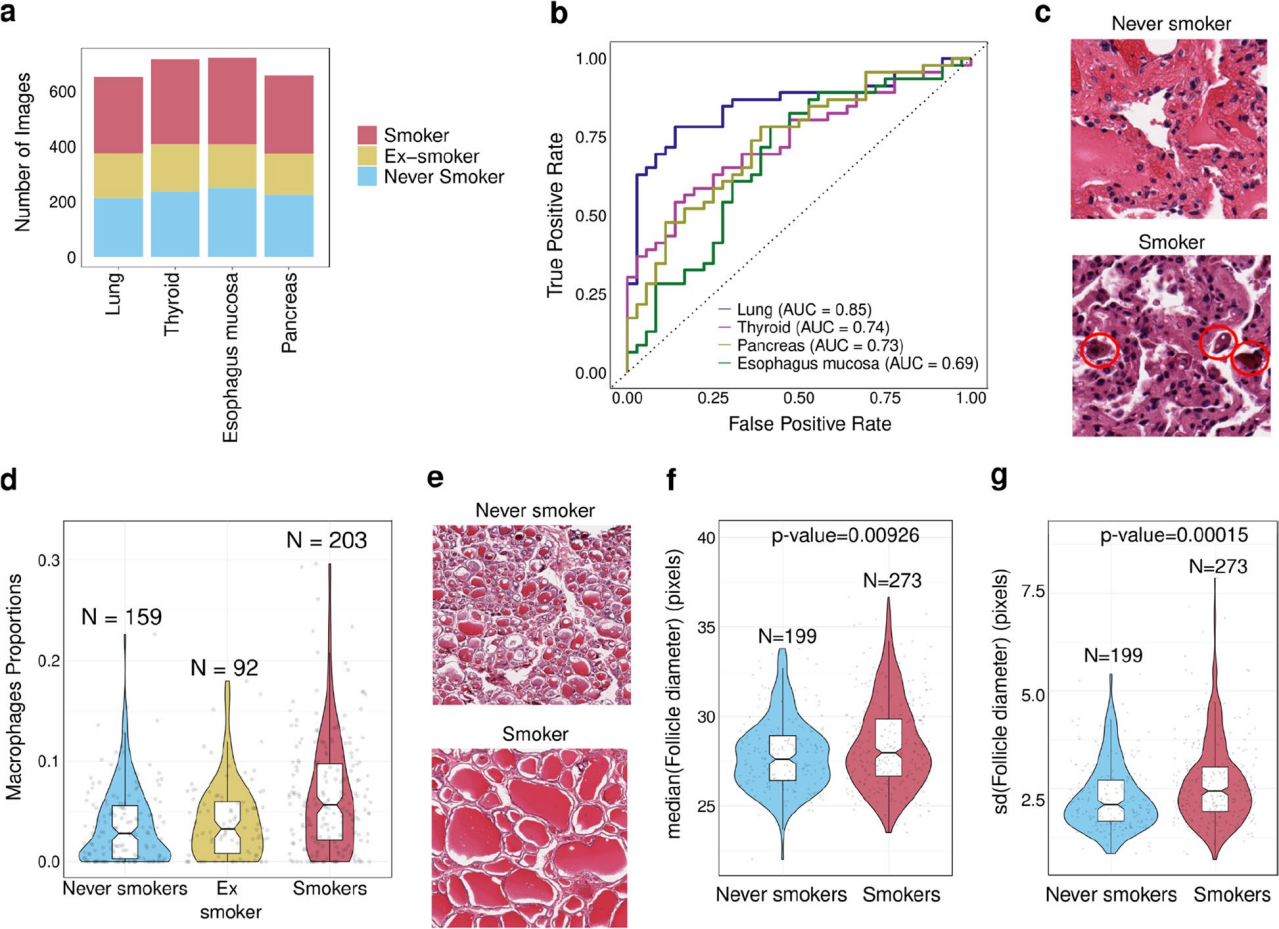
Impact of smoking on histology. **a** Number of images analyzed per tissue and per donor smoking status. **b** Receiver operating characteristic (ROC) curves of the tissue histology classifiers. **c** Example images of a never smoker and a smoker’s lung. Brown macrophages are highlighted in red circles. **d** Estimated proportion of macrophages in never smokers, ex-smokers, and smokers. **e** Example images of a never smoker and smoker’s thyroid. Smoker thyroids have larger colloid-containing follicles. **f** Mean (per donor) of median (per tile) diameter of thyroid follicles. *p*-value obtained from a Wilcoxon test. **g** Median (per donor) of standard deviation (sd) (per tile) diameter of thyroid follicles. *p*-value obtained from a Wilcoxon test

We hypothesized that the observed smoking-induced gene expression effects could be in part explained by histological changes in those tissues. To further investigate the impact of smoking on tissue architecture, we selected the four tissues with the largest number of smoking-DEGs (Fig. 1a: lung, thyroid, pancreas, and esophagus mucosa) and analyzed their histological images (Fig. 3a). We trained a convolutional neural network per tissue to classify histology images as smokers or never smokers (Additional file 3: Fig. S3a). All classifiers have higher performance than expected by chance (Fig. 3b), suggesting that smoking has an impact on the four tissues’ histology. The lung classifier has the best performance (AUC = 0.85), consistent with the known damaging effects of smoking in this tissue [92–95]. Visual inspection of the lung images revealed a substantial presence of macrophages (Fig. 3c). Cell type deconvolution analysis (Methods) confirmed that smokers have a higher proportion of macrophages (*z*-test; FDR = 2.26e − 06), according to previous reports [92–94] (Fig. 3d, Additional file 8: Table S7).

The thyroid classifier has the second-best performance (AUC = 0.74), consistent with thyroid being the second tissue with the largest number of smoking-DEGs. We visually inspected thyroid images and observed that smokers have bigger colloid-containing follicles (Fig. 3e). Thyroid follicles are the storage unit of inactive thyroid hormones. We used *CellProfiler * [50] to identify thyroid follicles automatically from the images and compute size and shape metrics (Additional file 3: Fig. S3b, Additional file 9: Table S8). We find that smokers have larger mean (Fig. 3f) and standard deviation (Fig. 3g) follicle diameters compared to never smokers. This is consistent with the well-known association between smoking and goiter, which is an irregular growth of the thyroid gland [85]. Pancreas and esophagus mucosa models show similar discriminatory performance, highlighting that cells and tissues undergo structural changes as a result of smoking.

### Smoking and aging alter gene expression across tissues in the same direction

**Fig. 4:**
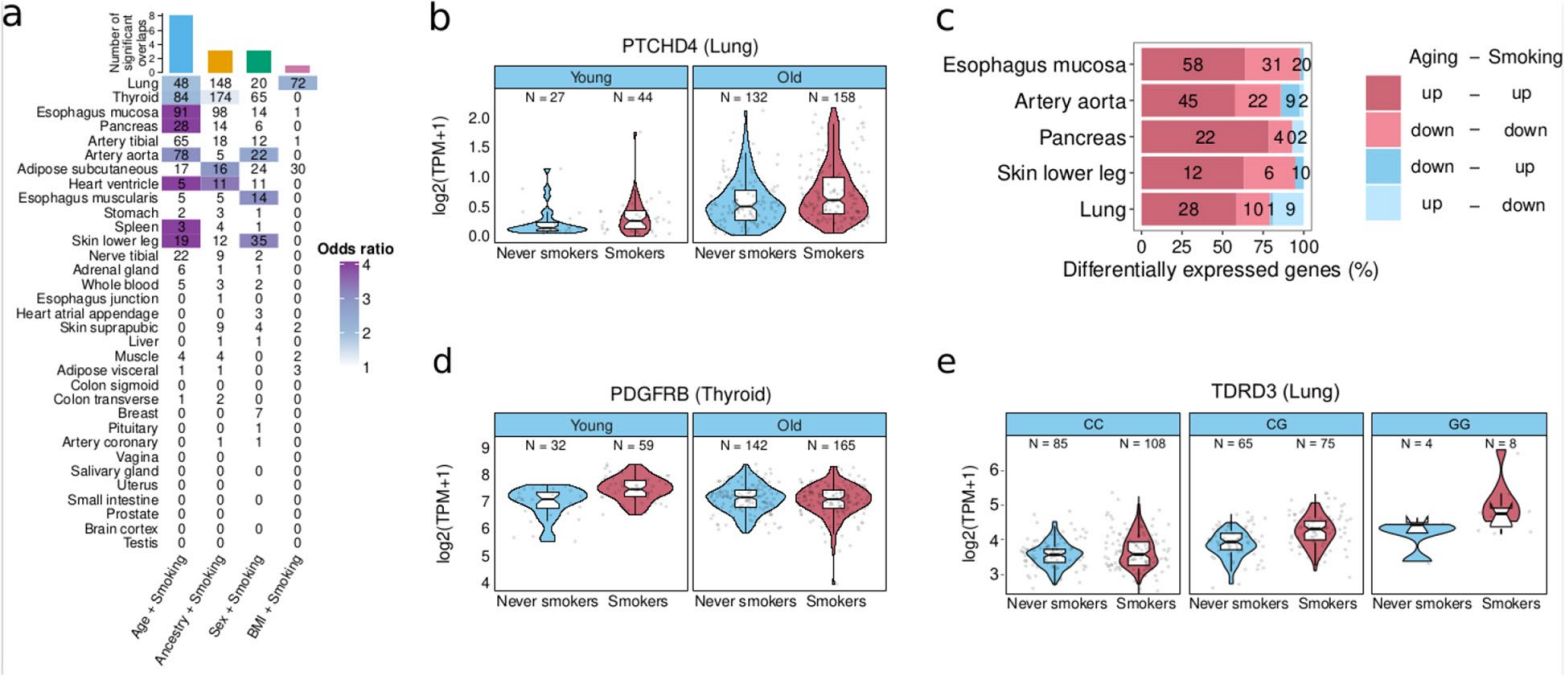
Impact of smoking and aging on gene expression. **a** Number of smoking-DEGs that are also DE with one demographic trait per tissue. Colored cells indicate higher-than-expected overlaps (Fisher’s exact tests; FDR < 0.05). Tissues are sorted from highest (top) to lowest (bottom) sample size. **b** Example of a gene with additive effects for smoking and age. **c** Tissues with significant concordance in the direction of change (up- or down-regulation) for smoking-age-DEGs across tissues (chi-squared tests; FDR < 0.05). Tissues are sorted from lowest (top) to highest (bottom) *p*-value. **d** Example of a gene with an interaction effect between smoking and age. **e** Expression of *TDRD3* stratified by genotype at rs7924558 position and smoking status

Common gene expression changes between smoking and aging in the respiratory tract have suggested that smoking could be an aging accelerator [12, 13]. To assess if this extends to other tissues, we tested if the overlap between smoking-DEGs and age-DEGs is higher than expected by chance. For comparison, we performed the same test on three other demographic traits: sex, ancestry, and body mass index (BMI), which are also known to influence gene expression variation [32]. Age is the demographic trait with the largest number of tissues with significant overlap with smoking-DEGs, with 8 tissues showing more smoking-age-DEGs (genes that are smoking-DEGs and age-DEGs) than expected by chance (Fig. 4a–b) (one-tailed Fisher’s exact test; FDR < 0.05). For the 6 tissues with more than 10 smoking-age-DEGs, 5 of them have a significant bias in the direction of change (chi-squared test; FDR < 0.05). In all cases, the direction of the effect is concordant between smoking and age (Fig. 4c, Additional file 3: Fig. S4a–c). We find significantly enriched pathways for the concordant smoking-age-DEGs in four tissues, with the strongest associations related to immune functions for upregulated genes in the pancreas and skin (Additional file 10: Table S9).

We then tested if the effects of smoking differed depending on intrinsic donor characteristics, such as age or genetic background. First, we performed differential gene expression analysis, extending the previous models by adding an interaction term for smoking and each of the four demographic traits (age, sex, genetic ancestry and BMI). We identified 10 genes with significant interactions (Additional file 3: Fig. S4 d). For instance, *PDGFRB,* a gene involved in premature aging [96], is upregulated in young smokers (Fig. 4d). Then, to test whether the impact of smoking on gene expression varied depending on *cis*-genetic effects, we performed expression quantitative trait loci analysis (eQTL) across tissues, including a genotype × smoking interaction term (Methods). We identified one significant interaction in *TDRD3*, a chromatin remodeler gene, at position *rs7924558* in lung (Fig. 4e).

Overall, we find that smoking acts additively with aging, changing expression across tissues in the same direction, supporting the idea that smoking acts as a systemic tissue aging accelerator, often by increasing inflammation.

### Smoking induces hypermethylation at targets of the Polycomb repressive complex

**Fig. 5:**
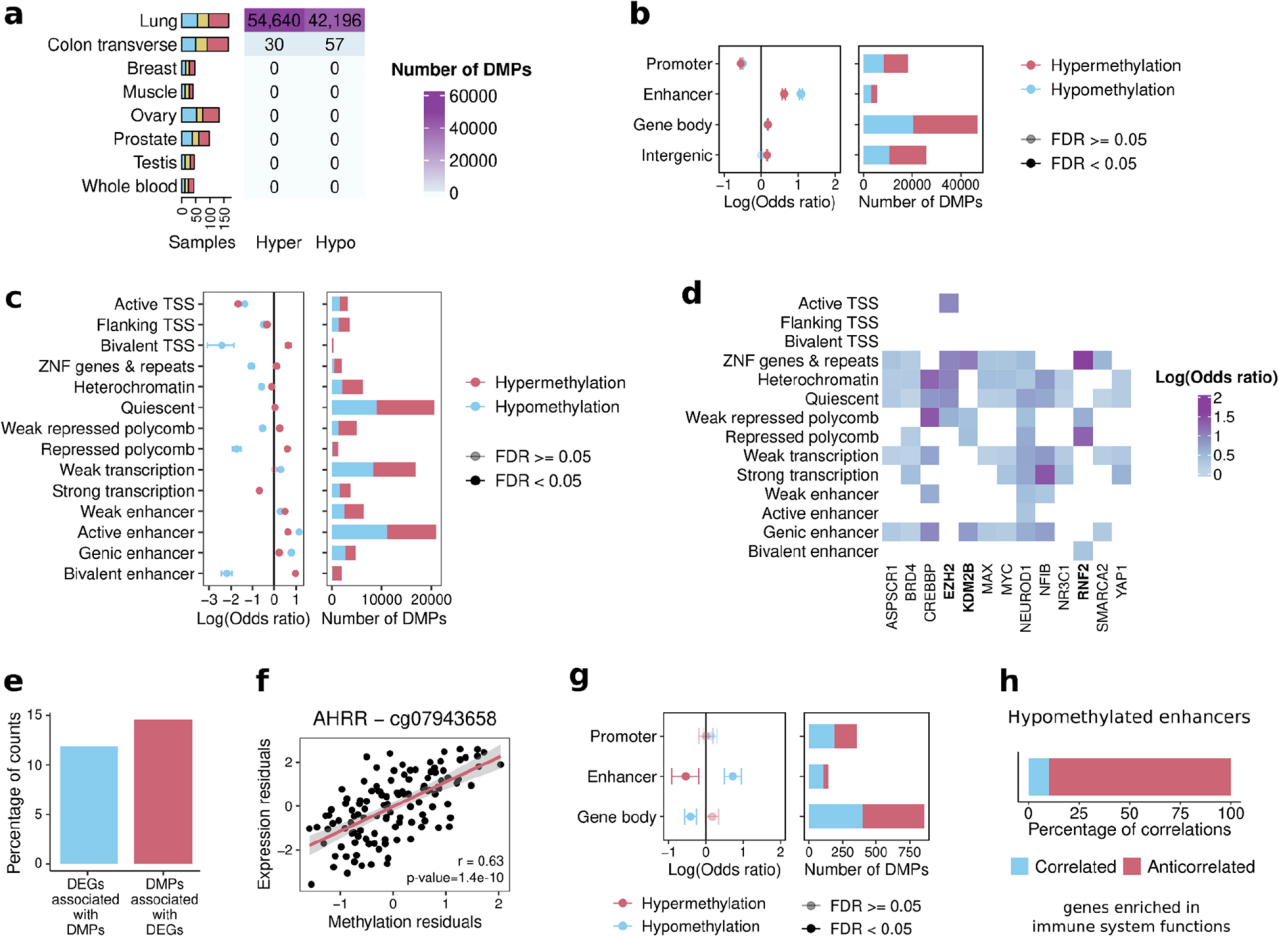
Association of smoking with DNA methylation. **a** Number of samples (left) and smoking-DMPs (right) across tissues. **b** Smoking-DMPs enrichment at regulatory regions. **c** Smoking-DMPs enrichment at chromatin states **d** TFBSs enriched in hypermethylated smoking-DMPs in more than 3 chromatin states. **e** Percentage of smoking-DEGs associated with at least one smoking-DMP, and vice versa. **f** Correlation of methylation residuals in cg25648203 with expression residuals of AHRR. **g** Enrichment of significantly correlated DMPs-DEGs in lung at regulatory regions. **h** Percentage of positive and negative correlations for hypomethylated enhancers (top), and the pathway in which these correlations are enrichment (bottom)

A recent study generated DNA methylation data for 9 GTEx tissues and showed that smoking is associated with DNA methylation changes in the lung and colon [18]. We wanted to address if smoking-induced DNA methylation changes were similar to age-associated methylation changes, similarly to what we have observed in gene expression in the same cohort. To address this, we performed differential methylation analysis with smoking, correcting for two PEER factors across the 9 tissues (Additional file 3: Fig. S5a) (Methods). We identified 96,826 differentially methylated positions (smoking-DMPs) in the lung and 87 in the colon transverse (Fig. 5a, Additional file 11: Table S10) [18]. Smoking-DMPs significantly overlap those identified in previous studies (Additional file 3: Fig. S5b; Fisher’s exact tests; FDR < 0.05) [17, 18, 57–61]. However, we identify a larger number of smoking-DMPs in the lung and a higher proportion of hypermethylation compared to other studies [17, 18, 57, 58, 60, 61, 97]. This higher proportion of hypermethylation might be due to our analysis detecting DMPs with smaller effect sizes that cannot be detected with lower sample sizes. Indeed, hypomethylated CpGs have larger effect sizes (Additional file 3: Fig. S5c) and when downsampling to smaller sample sizes, we retrieve higher proportions of hypomethylation (Additional file 3: Fig. S5 d), suggesting that they are easier to detect.

We then characterized our newly identified smoking-DMPs according to genomic context because DNA methylation can have distinct regulatory effects when occurring in different genomic regions [98]. In lung, smoking-DMPs are depleted in promoters and CpG islands and enriched in enhancers and open sea regions (Fig. 5b, Additional file 3: Fig. S5e–f). Using lung-specific chromatin segmentations as defined by ChromHMM [64], we find bivalent enhancers, bivalent TSS, and Polycomb-repressed regions enriched in hypermethylated smoking-DMPs (OR = 2) and depleted in hypomethylated smoking-DMPs (OR = 0.25, Fig. 5c). When looking at genes having smoking-DMPs nearby, we observe these genes are mostly associated with developmental functions, with the strongest signal in hypermethylated smoking-DMPs at active enhancers, active and flanking TSS, and quiescent regions (Additional file 12: Table S11). Whereas enrichment of smoking-DMPs at enhancers has been previously observed [57, 61], the observation that hypermethylated smoking-DMPs are enriched at bivalent and Polycomb-repressed regions is novel. These regions have in common low basal levels of methylation (Additional file 3: Fig. S6a) and are marked by the repressive histone mark H3 K27 me3 placed by the Polycomb repressive complex [63]. From now on, we will refer to the combination of bivalent TSS, bivalent enhancer, repressed polycomb, and weakly repressed polycomb chromatin states as Polycomb targets. Consistent with this, among the transcription factors (TFs) enriched in hypermethylated smoking-DMPs and shared across most chromatin regions (Fig. 5d, Additional file 3: Fig. S6 d, Additional file 13: Table S12) are TFs that are part of the Polycomb repressive complex (*EZH2*, *RNF2*, and *KDM2B*).

To check if the effect of smoking on Polycomb targets extends to other tissues, we looked at the location of DMPs reported in a whole blood study with a larger sample size [61]. Hypermethylated DMPs are significantly enriched in bivalent TSS (Additional file 3: Fig. S6b) (Fisher’s exact test; OR = 4, FDR = 0.029). The second-highest odds ratio, although marginally not significant, was at bivalent enhancers (Fisher’s exact test; OR = 2.3, FDR = 0.06). Furthermore, analysis of a whole-genome bisulfite dataset [72] including six tissues (Additional file 3: Fig. S6c), replicates the enrichment of hypermethylation at Polycomb targets (Additional file 3: Fig. S6 d) both in the lung and in the brain amygdala, and the enrichment of hypermethylation at CpG islands in the lung and three brain tissues (Additional file 3: Fig S6e). This suggests that smoking-induced hypermethylation at Polycomb targets is shared across tissues.

We then wanted to explore if the observed smoking-induced methylation correlates with gene expression changes. Restricting our analysis to CpG-gene pairs described in the EPIC annotation (Methods), we observe very few (11–15%) DMP-DEG pairs (Fig. 5e–f) suggesting that, in most cases, smoking has independent effects on DNA methylation and gene expression. In addition, from the DMP-DEGs, only 12.6% of the smoking-DMPs (1356) were correlated with the associated smoking-DEG, mostly negatively (68%, Additional file 3: Fig. S7a). Interestingly, the correlated smoking-DMPs in the lung are enriched in hypomethylated enhancers (Fig. 5g), where 90% of the correlations are negative (Fig. 5h). These hypomethylated enhancers are correlated with genes enriched in functions related to the immune system (Additional file 14: Table S13), suggesting that previously observed smoking-induced DNA hypomethylation at enhancers [57, 61] can be related to immune response. Interestingly, hypermethylated CpGs at Polycomb targets show fewer significant correlations with gene expression (4%) than other hypermethylated positions with similar effect sizes (15%) (binomial test; *p*-value = 5.16e − 08), suggesting that smoking-induced hypermethylation at targets of the Polycomb repressive complex does not significantly impact gene expression.

To gain a more comprehensive understanding of the interplay between smoking-induced DNA methylation and gene expression changes, we applied Multi-Omics Factor Analysis (MOFA), a dimensionality reduction method designed for early integration of multi-omic datasets to identify latent factors capturing shared sources of variation [70]. We treated CpGs in different genomic contexts as distinct data modalities (Methods). In lung tissue, MOFA identified nine latent factors, of which only one (Factor 2) was significantly correlated with smoking status (cor = 0.45, FDR = 9.9e − 05, Additional file 3: Fig. S7b). This latent factor explains a major percentage of methylation variation, in particular at enhancers (13%) and promoters (9%), and to a lower extent, gene expression variation (2%, Additional file 3: Fig. S7c).

To further interpret the biological significance of Factor 2, we performed gene set enrichment analysis on the factor weights (Methods). This revealed that genes positively correlated with smoking were enriched for immune system-related terms (Additional file 3: Fig. S7 d). Interestingly, terms such as “immune response,” “B cell proliferation,” and “defense response” were also enriched in negatively correlated promoter- and enhancer-associated CpGs. The shared enrichment between gene expression and methylation signals reinforces our previous observation that smoking-induced hypomethylation at enhancers may be linked to immune response activation, while suggesting a similar regulatory association at promoters.

Finally, we wondered whether the differences in methylation could be produced by changes in the expression of methyltransferases, in charge of CpG methylation, or the TET enzymes, in charge of the CpG demethylation. However, we do not find any of these genes differentially expressed in this dataset (Additional file 4: Table S3).

Overall, we find that smoking drives hypomethylation in the regulatory elements of immune genes, coupled with their upregulation in gene expression. Furthermore, we report hypermethylation at target regions of the Polycomb repressive complex, although with marginal effects in gene expression.

### Smoking shows additive effects with aging at Polycomb binding sites and alters aging driver CpGs

**Fig. 6:**
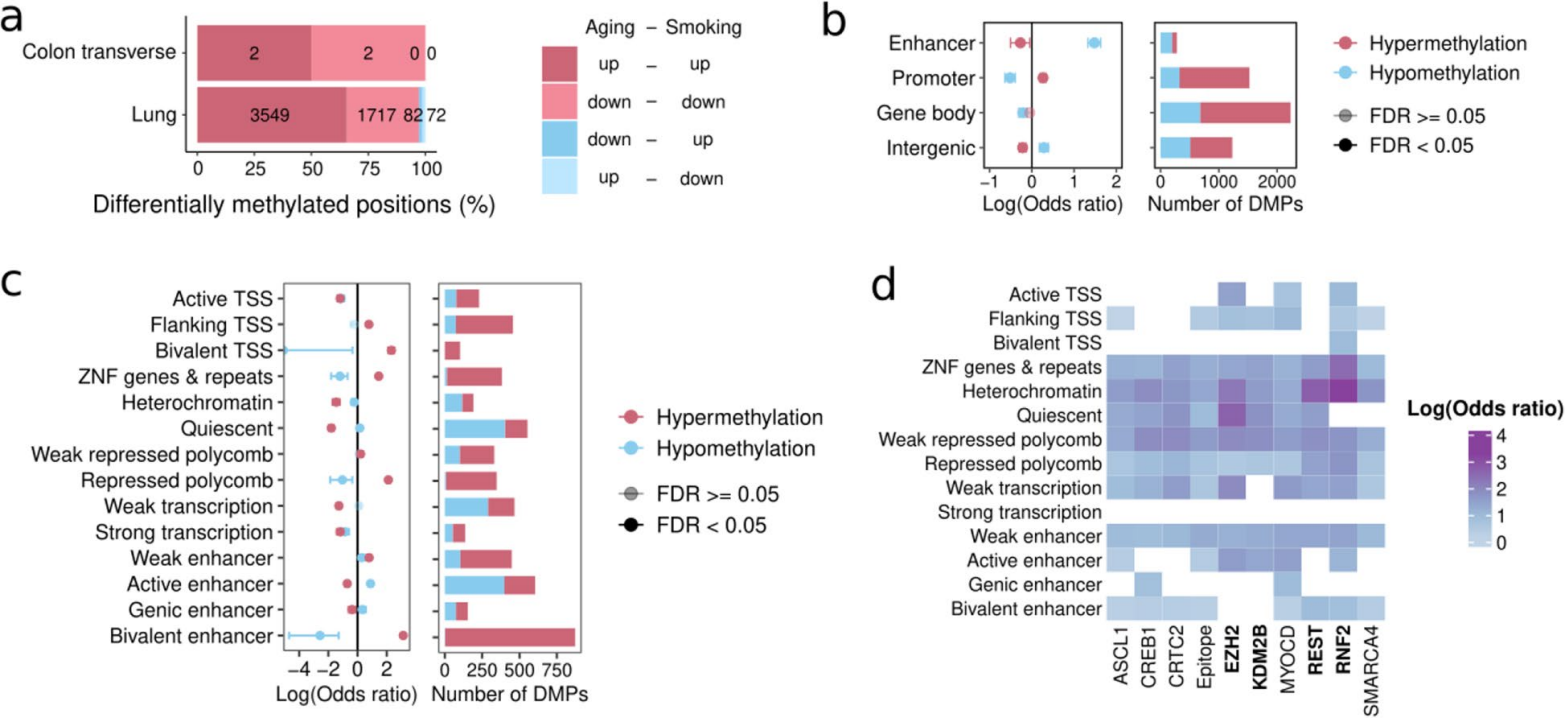
Smoking and age DNA methylation effects. **a** Tissues with significant concordance in the direction of change (up- or down-regulation) for smoking-age-DMPs (chi-squared tests; FDR < 0.05). **b** Enrichment of smoking-age-DMPs at regulatory regions. **c** Enrichment of smoking-age-DMPs at chromatin states. **d** Shared TFBSs enriched in hypermethylation across more than 7 chromatin states for smoking-age-DMPs. TFs highlighted in bold are part of the Polycomb repressive complex

Cigarette smoking has been associated with increased epigenetic aging, as age-predictive models trained on methylation marks predict smokers as older than their chronological age [14, 15, 68]. Our analysis shows higher hypermethylation at bivalent and Polycomb-repressed regions (Fig. 5c), which is known to occur also in aging [99]. Thus, we tested whether smoking and aging impact the same CpGs in the same direction across tissues. We find that smoking-DMPs significantly overlap age-DMPs in the same direction in both lung and colon (Fig. 6a) (chi-square test; FDR < 0.05). The strongest enrichment of smoking-age-DMPs (CpGs that are smoking-DMPs and age-DMPs) occurs at hypomethylated enhancers (Fig. 6b) and open sea regions (Additional file 3: Fig. S7e). When we focus on chromatin states, the strongest enrichments occur for hypermethylation in bivalent enhancers (25-fold), bivalent TSS (tenfold), and Polycomb-repressed regions (tenfold) (Fig. 6c), regions associated with development that are known to be hypermethylated with aging [62, 100]. Functional enrichment analysis on the genes associated with smoking-age-DMPs shows that hypermethylation in active TSS is also associated with developmental genes (Additional file 12: Table S11).

We then explored the enrichment of smoking-age-DMPs in TFBSs. We find TFs enriched in almost all chromatin states (Additional file 3: Fig. S7f). From the most shared TFs in hypermethylated smoking-age-DMPs, we find four components of the Polycomb repressive complex (EZH2, RNF2, KDM2B, and REST) enriched in almost all chromatin states (Fig. 6d, Additional file 3: Fig. S7 g, Additional file 13: Table S12). Hence, both smoking and aging induce similar changes in DNA methylation, particularly through hypermethylation at targets of the Polycomb repressive complex.

A recent study employed an epigenome-wide Mendelian randomization approach to identify causal CpG for aging-related phenotypes [68, 78]. This approach leverages genetic variants to test whether a biomarker influences a trait through a causal pathway rather than mere correlation [101]. Leveraging these results, we wondered if smoking-induced DNA methylation changes could directly cause tissue aging. We first assessed the degree of overlap between smoking-DMPs with causal aging CpGs and found that smoking-DMPs significantly overlap causal aging CpGs in lung (Additional file 3: Fig. S8a; Fisher’s exact test; FDR < 0.05). We then tested if smoking-DMPs were specifically enriched in either protective or damaging DNA methylation changes. Briefly, a protective methylation change is defined as a DNA methylation change that contributes to a positive impact on healthy longevity, while the opposite is true for damaging methylation change [68]. We find that smoking-DMPs are enriched in all four types of causal aging CpGs (protective hypomethylation, protective hypermethylation, damaging hypomethylation and damaging hypermethylation) with comparable effect sizes (Additional file 3: Fig. S8b–c; Fisher’s exact test; FDR < 0.05). However, combined damaging and protective DNA methylation changes have overall negative effects [68]. Thus, our analysis suggests that cigarette smoking accelerates tissue aging by modifying aging causal CpGs through its impact on DNA methylation, indicating a causal relationship rather than mere correlation.

Next, we evaluated whether DNA methylation clocks capture smoking-associated acceleration of biological aging across tissues. Specifically, we tested the Horvath pan-tissue clock [78] and two recently developed clocks based on causal CpGs for aging phenotypes: DamAge and AdaptAge, which are built on damaging and protective methylation sites, respectively [68]. After adjusting for technical and demographic covariates, we found no significant differences in predicted age between smokers and non-smokers using the Horvath clock in any tissue (*T*-test, FDR > 0.05, Additional file 3: Fig. S9a). Although a previous study [68] reported an association between the Horvath clock and smoking status in lung tissue, their analysis included tumor samples, which may explain the discrepancy. Our findings are consistent across multiple tissues and align with other studies that have not observed robust associations between smoking and Horvath clock predictions [102, 103]. In contrast, analysis of causal-CpG clocks revealed that smokers exhibited significantly higher predicted age in lung tissue using AdaptAge (*T*-test, FDR = 0.02, Additional file 3: Fig. S9b), suggesting accelerated biological aging due to altered methylation at protective CpG sites. No significant differences were observed for DamAge predictions across tissues (FDR > 0.05, Additional file 3: Fig. S9c). Together, these findings suggest that smoking may promote aging acceleration in the lung via perturbation of protective methylation marks, as captured by the AdaptAge clock.

### Smoking-induced DNA methylation changes shared with aging are more persistent

**Fig. 7:**
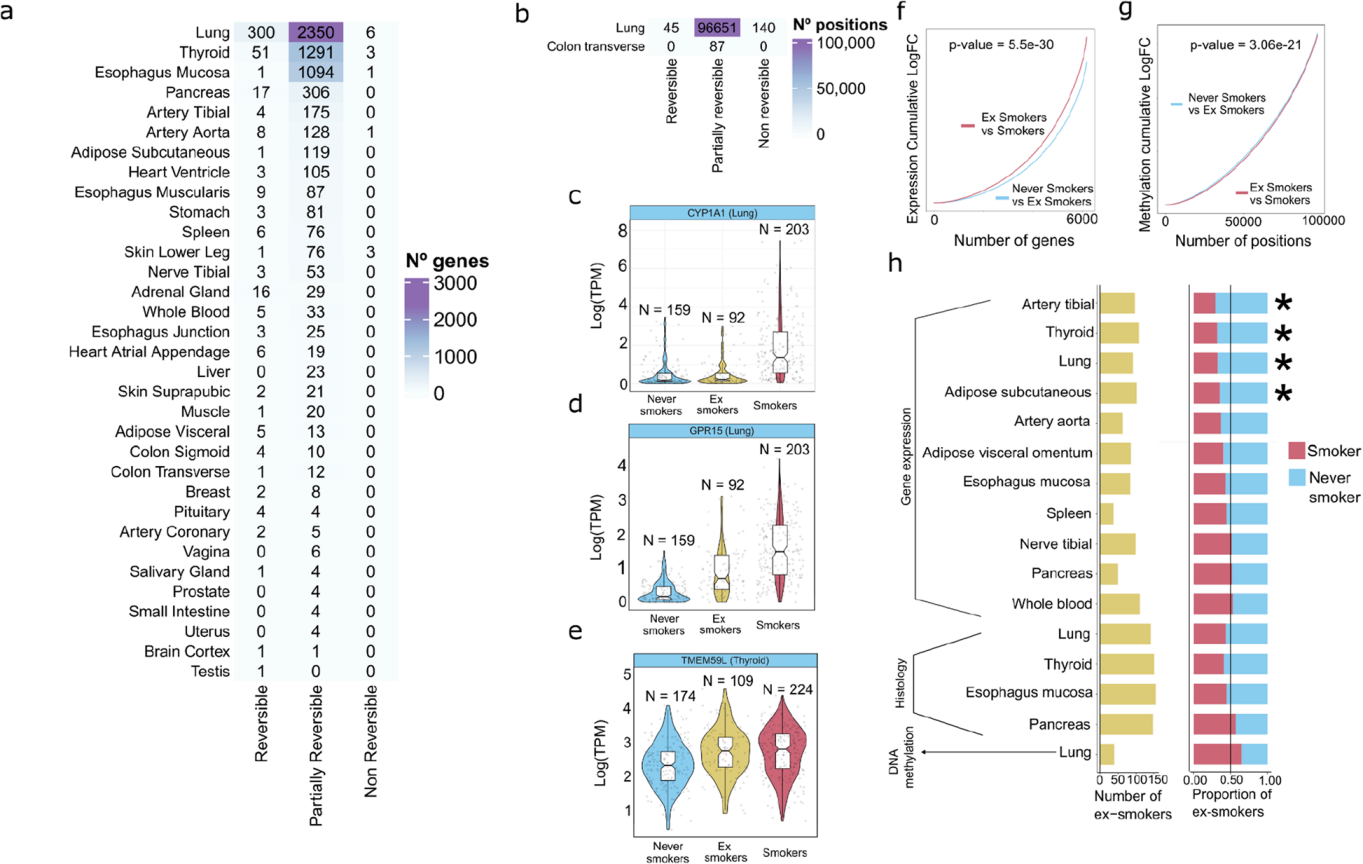
Molecular and histological impact of smoking cessation.** a** Smoking-DEGs classification into reversible, partially reversible, or non-reversible genes per tissue. **b** Smoking-DMP classification into reversible, partially reversible, and non-reversible. **c** Examples of a reversible gene, **d** a partially reversible gene, and **e** a non-reversible gene. **f** Smoking-DEGs log fold change in never vs. ex-smokers and in ex-smokers vs. smokers. *p*-value obtained from a Wilcoxon test. **g** Smoking-DMP log fold change in never vs. ex-smokers and in ex-smokers vs. smokers. *p*-value obtained from a Wilcoxon test. **h** Classification of ex-smoker individuals into smokers or never smokers per tissue in gene expression, histology, and methylation. The green bars represent the number of re-classified ex-smoker samples. For gene expression, only highly accurate models (AUC > 0.85) were considered for the classification of ex-smokers. Asterisks correspond to FDR < 0.05 for binomial tests

Smoking cessation is associated with decreased disease risk and improved life quality [104, 105]. Earlier studies examining gene expression and DNA methylation of the respiratory tract and whole blood in ex-smokers show that while the majority of smoking-induced changes revert after quitting, certain changes persist for years [34, 38, 60]. To explore the impact of smoking cessation across human tissues, we performed differential gene expression, alternative splicing, and DNA methylation analyses between ex-smokers and smokers, and between ex-smokers and never smokers (Additional file 3: Fig. S10a–b). We then classified smoking molecular perturbations as reversible, partially reversible, or non-reversible based on whether they are differentially expressed, spliced, or methylated between smokers and ex-smokers, between ex-smokers and never smokers, or neither (Methods, Fig. 7a–e, Additional file 3: Fig. S10c, Additional file 15: Table S14, Additional file 16: Table S15). The large majority of genes (92.86%, Fig. 7a), splicing events (96%, Additional file 3: Fig. S10c), and CpGs (99.8%, Fig. 7b) were classified as partially reversible. However, in gene expression, there were more reversible genes (6.92%) than non-reversible (0.21%). Conversely, for DNA methylation, there were less reversible CpGs (0.05%) compared to non-reversible CpGs (0.14%). Consistent with this, ex-smokers have more partially reversible genes (57%) closer in expression to never smokers (Fig. 7f) but more partially reversible DMPs (52%) closer in methylation levels to smokers (Fig. 7g). Restricting gene expression and DNA methylation analysis to the same donors shows consistent results (Additional file 3: Fig. S10 d–e). This suggests that ex-smokers are more similar to never smokers in gene expression and to smokers in DNA methylation. To further test this, we trained machine learning classifiers of smokers and never smokers using either gene expression or DNA methylation (Additional file 3: Fig. S11a) and used them to reclassify ex-smokers. Gene expression models classify ex-smokers more often as never smokers, with statistically significant differences in lung, artery tibial, thyroid, and adipose subcutaneous (Fig. 7h; binomial test; FDR < 0.05). The DNA methylation model classifies 68% of ex-smokers as smokers (Fig. 7h; binomial test; *p*-value = 0.08). This provides further evidence that ex-smokers are more similar to never smokers in gene expression but more similar to smokers in DNA methylation.

Finally, we wanted to address whether smoking effects that overlap those of aging were more or less persistent in time compared to those that do not. First, we tested if reversible, partially reversible, or non-reversible smoking-DEGs significantly overlap age-DEGs. We find one significant overlap between non-reversible smoking-DEGs and age-DEGs in the skin, although the numbers are low (Fisher’s exact test; FDR = 0.0009). In DNA methylation, we find that non-reversible smoking-DMPs in the lung are significantly enriched in age-DMPs (Fisher’s exact test; *p*-value = 1.602e − 12), while the reversible and partially reversible smoking-DMPs are not. These results suggest that the smoking effects that affect DNA methylation in common to aging are more persistent in time.

Overall, our results suggest that smoking-induced DNA methylation alterations are more persistent than gene expression changes, especially those that overlap with aging.

## Discussion

Tobacco smoking is the primary cause of preventable mortality [1]. It has a systemic negative impact on health and increases the risk of developing many diseases [106]. Here, we provide an exhaustive catalog of molecular changes induced by cigarette smoking throughout the human body and report alterations in many previously unexplored tissues. The effects of smoking on gene expression primarily show tissue-specific effects rather than a shared gene signature, underscoring the importance of a tissue-by-tissue analysis. Nonetheless, we highlight a consistent upregulation of eight genes across multiple tissues. Three of these genes, *AHRR*, *CYP1 A1*, and *CYP1B1*, are pivotal components of the aryl hydrocarbon receptor signaling pathway, which is responsible for regulating xenobiotic metabolism [107], providing compelling evidence that tobacco toxins reach at least 30 human tissues and systematically activate this pathway to metabolize them. We also report systemic inflammation across tissues that can affect tissue architecture, as we show in the lung regarding macrophage infiltration [92]. However, we correct for most cell type composition changes in the gene expression analysis. Further work using single-cell data could address the impact of smoking on cell type composition and gene expression at particular cell types. Consistent with the reported inflammation (Fig. 2e), we find that the smoking-induced DNA methylation changes that correlate with gene expression are located at hypomethylated enhancers of immune-related genes. Interestingly, we report the thyroid as one of the most affected tissues by smoking and characterize its effects on tissue architecture. In particular, we find an enlargement of thyroid follicles, consistent with the association between smoking and an increase in the size of the thyroid gland, named goiter [85]. This could be caused by the presence of thiocyanate in cigarette smoke, which inhibits iodine uptake by the thyroid gland and might have a goitrogenic effect [85].

Our findings strongly support previous hypotheses that link smoking to accelerated aging [4, 5]. We provide evidence that smoking and aging similarly impact gene expression and DNA methylation across tissues. Specifically, genes associated with both traits are enriched in immune response pathways across tissues, which is in accordance with the well-established role of immune dysregulation during aging (inflammaging) [108]. Additionally, both smoking and aging drive hypermethylation of bivalent and Polycomb-repressed chromatin states. Importantly, we show that smoking-driven epigenetic changes in the lung are enriched in CpGs causal of aging, suggesting that smoking may in part directly mediate accelerated tissue aging through its effect on DNA methylation. Interestingly, while these age-causal CpGs were initially identified in blood [68], we observe the enrichment of age-causal CpGs in smoking-DMPs from the lung. This may further support the idea that smoking-driven aging acceleration might have a shared impact across tissues.

While it is well known that Polycomb target regions are hypermethylated with aging, the observation that this also happens in smokers is novel. Importantly, the biological basis for the known age-associated accumulation of DNA methylation in Polycomb target regions remains incompletely understood. One biological mechanism that could explain the hypermethylation at Polycomb target regions is mitotic turnover—DNA methylation errors that accumulate during cell division [109]. While this may explain age-related methylation in proliferative tissues, its relationship with smoking-induced changes, particularly in tissues with varying proliferative capacities, remains unclear and warrants further exploration. An alternative hypothesis involves persistent DNA damage and the recruitment of DNA methyltransferases during the repair process. Aging is associated with increasing DNA damage, and the repair machinery, containing DNA methyltransferases and the Polycomb repressive complex, appears to favor CpG-rich regions—including the ones we refer to as Polycomb targets—as substrates for de novo methylation [99, 110]. This model provides a plausible explanation for the systematic hypermethylation observed in these regions with age. Importantly, it aligns with our observation that smoking, a known source of systemic DNA damage [19], induces similar hypermethylation at both Polycomb targets and CpG islands (Additional file 3: Fig. S7e). According to this framework, the aging effects on Polycomb targets may reflect a mechanism driven by cumulative damage—whether from exogenous insults (e.g., tobacco toxins, UV exposure) or endogenous stressors (e.g., oxidative stress, inflammation).

There is ongoing debate on whether age-related epigenetic changes result from stochastic drift or regulated biological processes [111, 112]. The stochastic model proposes that DNA methylation changes arise randomly due to replication errors or incomplete maintenance, rather than from specific regulatory mechanisms. Mathematical models predicting stochastic changes can capture the methylation accumulation in lowly methylated regions and the loss of methylation in heavily methylated regions. While Polycomb targets generally exhibit low methylation and could fit the stochastic model, our finding that both smoking and aging produce similar patterns in this subset of loci supports a more deterministic mechanism that drives hypermethylation specifically, potentially triggered by DNA damage. Supporting this, DNA methylation clocks trained to predict biological age are less influenced by stochastic changes than those trained on chronological age [113]. Additionally, smoking-related aging acceleration is driven by nonstochastic processes rather than an increased rate of stochastic change [113]. Together, these observations suggest that methylation accumulation at Polycomb targets by both smoking and aging is orchestrated by a regulated biological process rather than random drift.

## Conclusions

Our study provides the most comprehensive, multi-tissue, and multi-omic analysis to date of the molecular effects of cigarette smoking, revealing that smoking mirrors and accelerates key aging processes across diverse human tissues. By integrating gene expression, alternative splicing, DNA methylation, and histological data, we demonstrate that smoking triggers systemic inflammation and leads to epigenetic alterations—particularly hypermethylation at Polycomb target regions—closely aligned with known aging mechanisms. Importantly, we identify some smoking-induced molecular changes that are not reversible after cessation, especially those overlapping with aging signatures. These findings not only advance our understanding of how smoking contributes to long-term tissue dysfunction and disease risk but also highlight molecular biomarkers that could inform personalized risk assessment, targeted prevention strategies, and the development of epigenetic-based interventions aimed at mitigating smoking-induced and age-related decline.

## Supplementary Information

Additional File 1: Table S1: Demographic information of the GTEx v8 dataset per tissue.

Additional File 2: Table S2: Clinical traits included per tissue.

Additional File 3: Fig. S1-S11.

Additional File 4: Table S3: Smoking-DEGs per tissue.

Additional File 5: Table S4: Enrichment analysis of all termsand all Ontologies.

Additional File 6: Table S5: Enrichment analysis—Orsum.

Additional File 7: Table S6: Smoking-DSEs per tissue. It includes a column with the most expressed isoform including and excluding the event and the protein domains where the event is involved for those isoforms.

Additional File 8: Table S7: Estimated cellular proportion of macrophages in the lung.

Additional File 9: Table S8: Features measured on thyroid follicles identified on thyroid histopathological images via CellProfiler.

Additional File 10: Table S9: Functional enrichments of smoking-age-DEGs.

Additional File 11: Table S10: DMPs per tissue.

Additional File 12: Table S11: Functional enrichments per chromatin region of smoking-DMPs, smoking-age-DMPs, smoking-DMPs without DMPs sitting in TFBSs from Polycomb proteins, and smoking-age-DMPs without DMPs sitting in TFBSs from Polycomb proteins.

Additional File 13: Table S12: TFBSs enrichment of smoking-DMPs and smoking-age-DMPs.

Additional File 14: Table S13: Functional enrichment of smoking-DEGs correlated with smoking-DMPs.

Additional File 15: Table S14: Never smokers vs ex-smokers and ex-smokers vs smokers expression analysis.

Additional File 16: Table S15: Never smokers vs ex-smokers and ex-smokers vs smokers methylation analysis.

## Data Availability

No datasets were generated or analysed during the current study. All GTEx protected data are available at dbGap under the accession number phs000424.v8: https://www.ncbi.nlm.nih.gov/projects/gap/cgi-bin/study.cgi?study_id=phs000424.v8.p2 [114]. Expression and histology data is publicly available through the GTEx Portal as downloadable files (https://gtexportal.org/home/histologyPage) [33]. DNA methylation data is available in GEO under the accession number GSE213478: https://www.ncbi.nlm.nih.gov/geo/query/acc.cgi?acc=GSE213478 [115]. The single-cell data is available in GEO under the accession number GSE173896: https://www.ncbi.nlm.nih.gov/geo/query/acc.cgi?acc=GSE173896 [116]. Analysis scripts are available at https://github.com/Mele-Lab/2024_GTEx_Smoking [117].
