# Supplementary material for: The molecular impact of cigarette smoking resembles aging across tissues
Source: Genome Med. 2025 Jun 2;17:66. doi: 10.1186/s13073-025-01485-x (PMC12131351; doi:10.1186/s13073-025-01485-x)
Supplement: Supplementary file 3 — Additional File 3: Fig. S1-S11. [file 13073_2025_1485_MOESM3_ESM.pdf]

Supplementary figures:

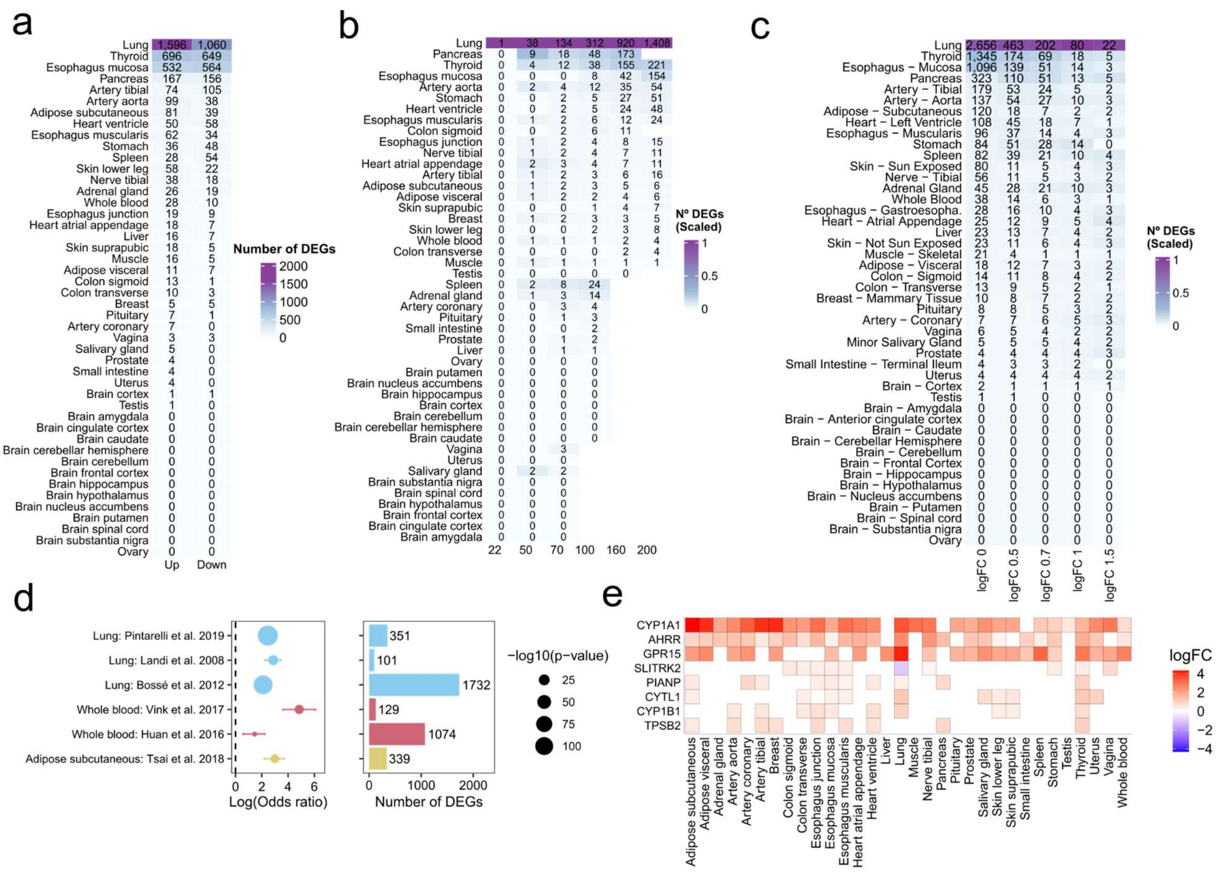

Fig. S1 - **Differential expression analysis. a**, Number of upregulated and downregulated smoking-DEGs per tissue. Tissues are sorted by total number of DEGs. **b**, Median number of smoking-DEGs per tissue when downsampling in 50 permutations. The x axis represents the different sample sizes, divided into equal numbers of smokers and never smokers. Tissues are sorted by number of DEGs with 160 samples **c,** Number of smoking-DEGs across tissues, filtered using different log fold change (logFC) cutoffs **d**, Replication of the smoking-DEGs found in this work with previous literature. The dot plots show the log odds ratio from the two-sided Wilcoxon-test, with respective confidence intervals, while the bar plot shows the number of DEGs in each study. Statistical significant overlaps were found with all studies. **e**, Log fold changes of the recurrent genes across tissues. A white square represents a tissue where that gene was not found differential expressed (FDR > 0.05).

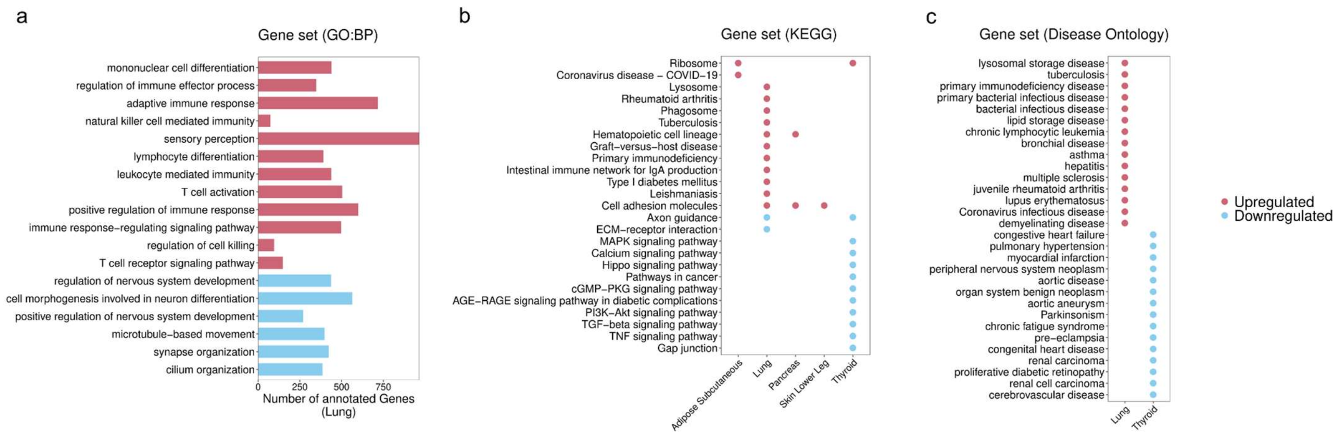

Fig. S2 - Enrichment analysis performed on smoking-DEGs across tissues. **a**, Top enriched gene ontology (biological process) in lung for upregulated (red) and downregulated (blue) genes in lung (FDR < 0.05 and minimum gene count > 5). **b**, Top enriched KEGG terms (FDR < 0.05 and minimum gene count > 5), for upregulated genes (red) and downregulated genes (blue) across tissues. Only the top 15 terms per tissue are represented. **c**, Top enriched disease ontology terms enriched in lung and thyroid (FDR < 0.05 and minimum gene count > 5).

a

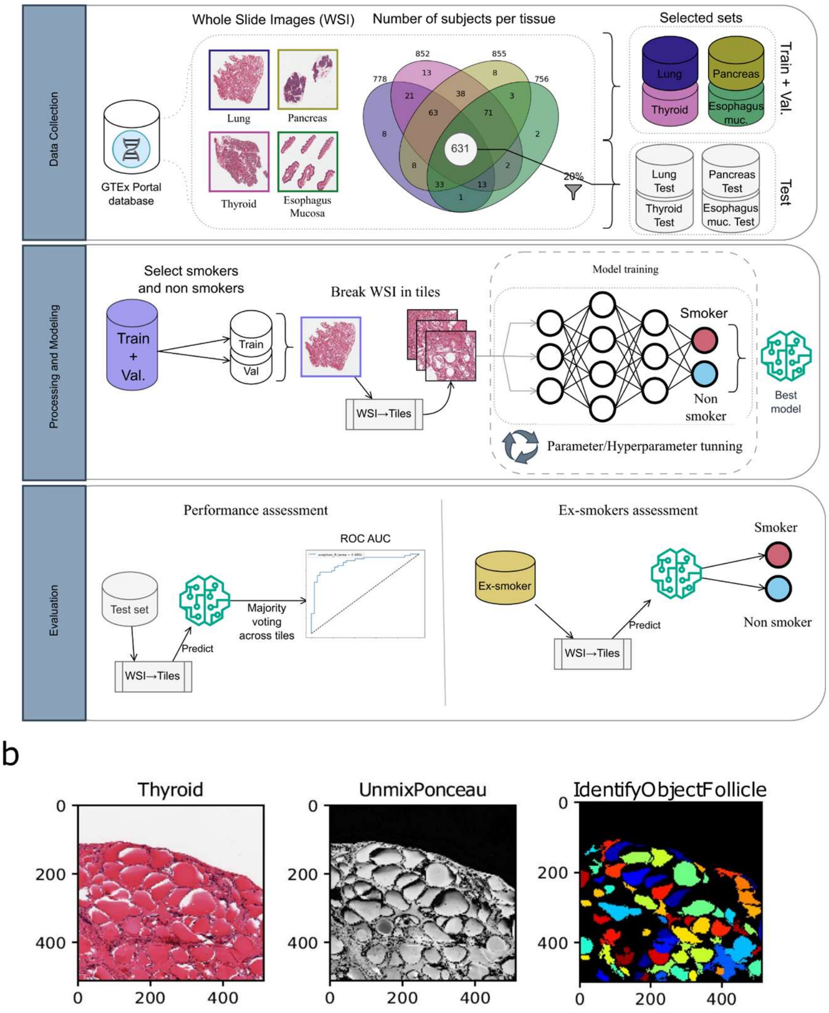

Fig. S3 - Histology Images analysis. a, General scheme of the machine learning approach used in this work for histology analysis. Whole slide images (WSI) for lung, thyroid, pancreas and esophagus mucosa were downloaded from the GTEx portal database. We split the data into train, validation (val) and test set. The test set was derived by taking 20% (126 subject) of the common samples across all tissues (631). The remaining samples from each tissue were further divided into train (80%) and validation (20%). For model training, WSI were divided into tiles, and kept tiles with at least 85% tissue content. For modeling, we used the pre-trained convolutional neural network (CNN) Xception changing only the top layers. Since the model outputs tile specific prediction, we used a majority voting approach to determine subject level prediction. The final model performance was evaluated in the test set. Finally, these models were used on ex-smoker samples to be classified as smoker or never smoker. **b**, CellProfiler pipeline illustration of a tile. Left — the original H&E stained tile of a thyroid, Center — Grayscale tile after computational transform with the Ponceau-Fuchsin stain, Right — Identified individual thyroid follicles (color).

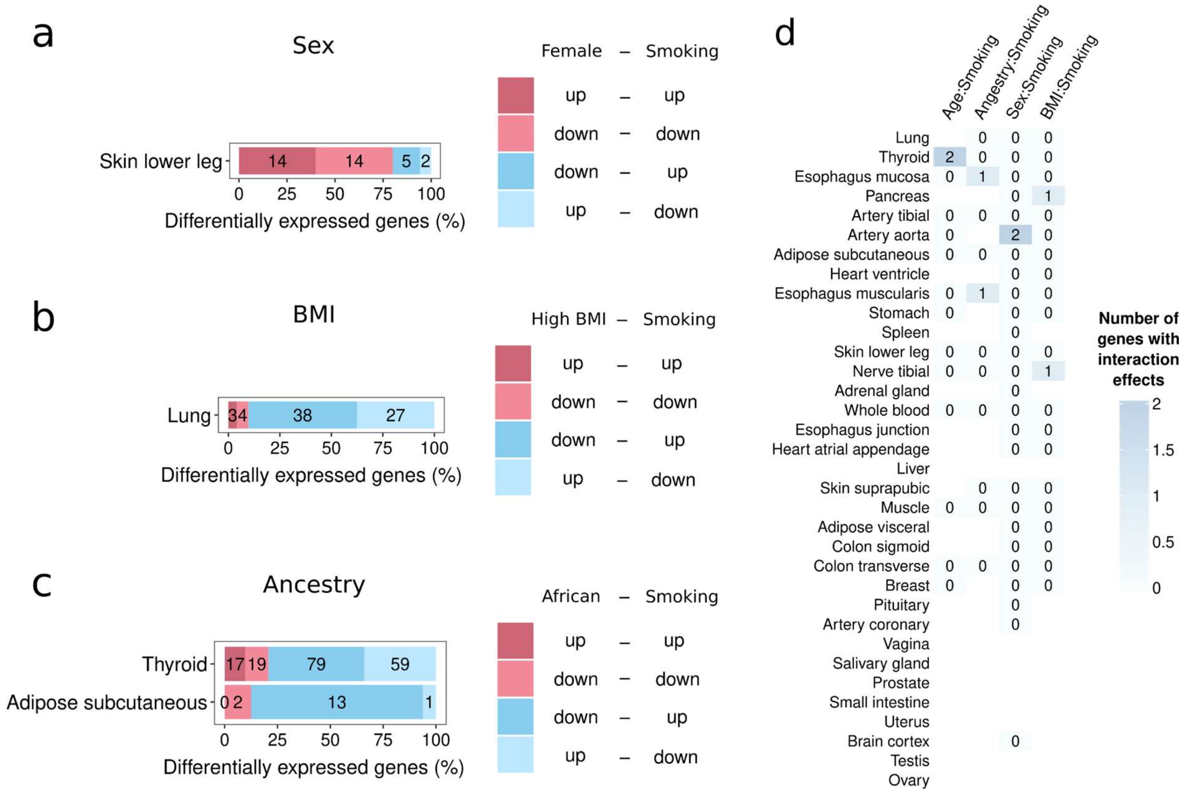

Fig. S4 - **Additive and interaction effects**. **a-c**, Bar plots showing the tissues with significant concordance in the direction of change (up- or down-regulation) for smoking-sex-DEGs, smoking-BMI-DEGs and smoking-ancestry-DEGs across tissues (chi-squared tests FDR<0.05). **d**, Genes with interaction effects between smoking and the different demographic traits.

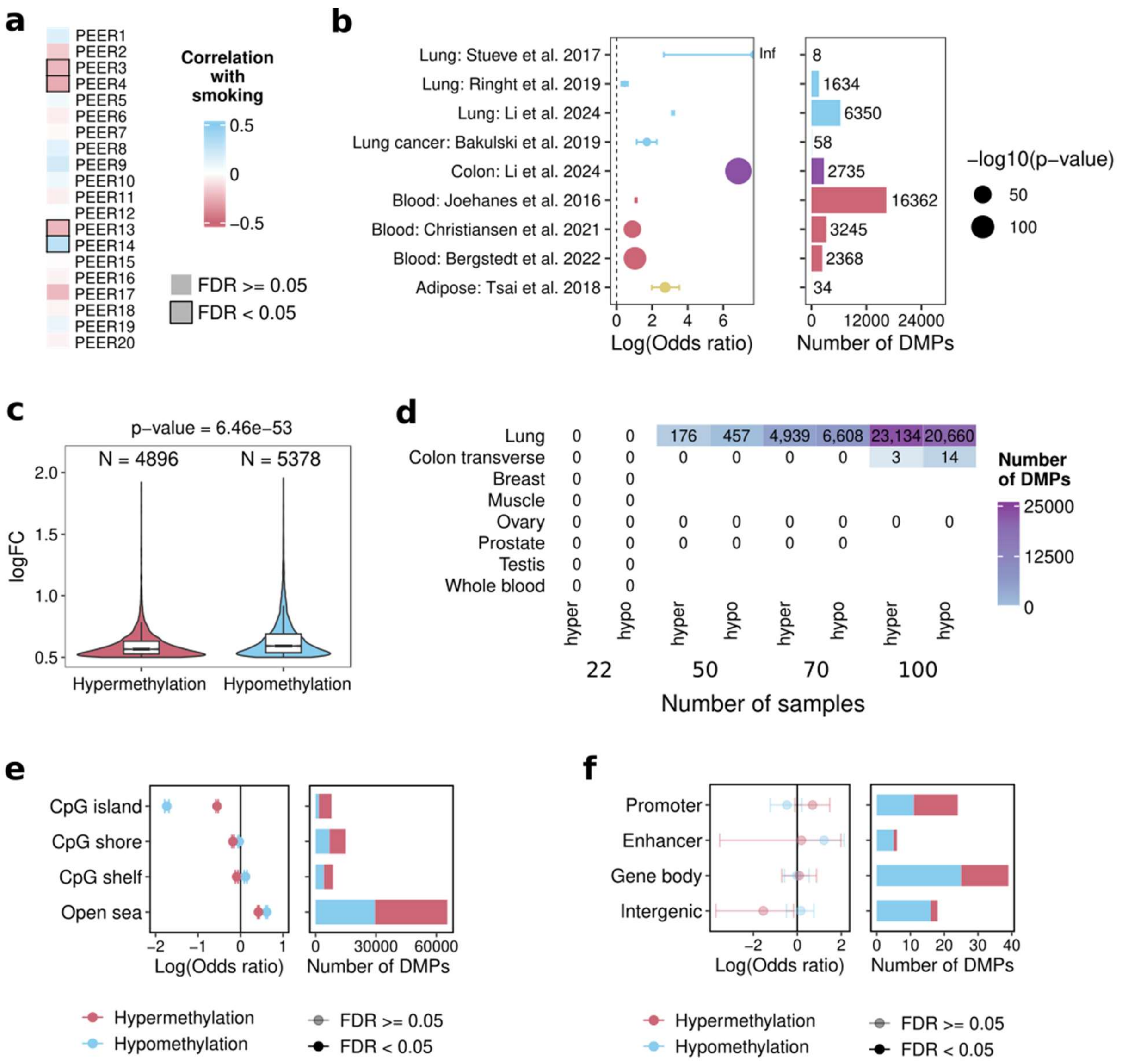

Fig. S5: Association between smoking and DNA methylation across tissues. a, Correlation between smoking and PEER factors. **b**, Replication of smoking-DMPs with DMPs identified in previous studies (Fisher's exact tests<0.05). The overlap in colon was performed with our colon DMPs, whereas all the rest were performed with our lung DMPs. Y-axis shows the article reference and the tissue used in the respective study. Left plot dashed line indicates odds ratio = 1, values above are enriched and below are depleted. Colors refer to the tissue used in the studies. Right plot shows the number of DMPs reported in each study that we can test. **c**, Effect sizes of hypermethylated and hypomethylated positions in lung. **d**, Number of DMPs obtained when downsampling to different number of samples (x-axis). The number of smokers and never smokers is the same. The cell numbers correspond to the mean of 50 different permutations per tissue and number of samples. **e**, Enrichment of smoking-DMPs on EPIC annotations about CpG context. **f**, Enrichment of colon transverse smoking-DMPs at regulatory regions.

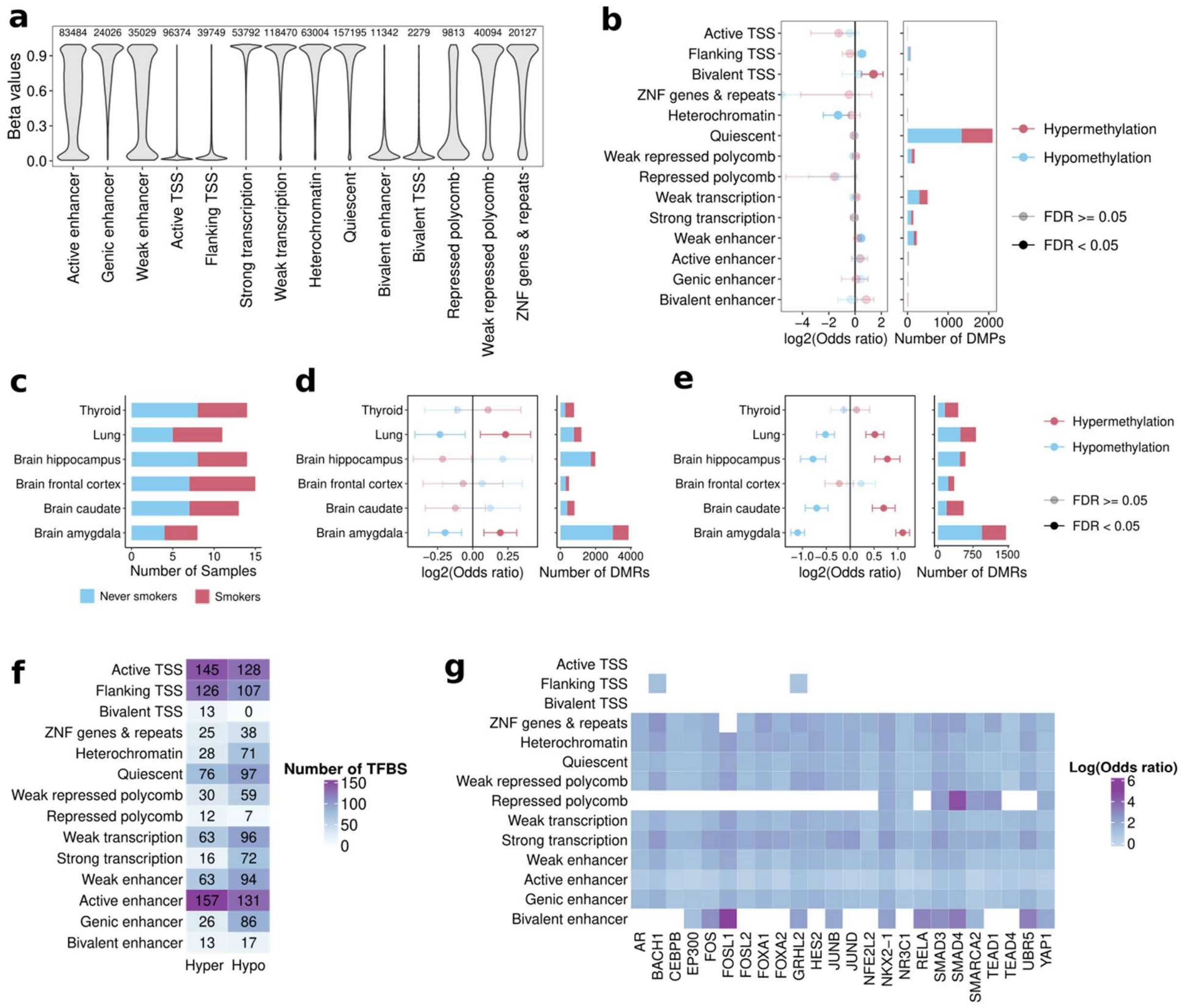

Fig. S6: Association between smoking and DNA methylation across chromatin states. a, Methylation levels in never smokers stratified by chromatin state. **b**, Enrichment of smoking-DMPs from Christiansen et al. at blood chromatin states. **c**, Number of samples for smokers and never smokers in the whole genome bisulfite sequencing dataset. **d**, Enrichment (left) and number (right) of smoking-DMRs in Polycomb targets (the overlap of bivalent TSS, weak repressed polycomb, repressed polycomb, and bivalent enhancer chromatin states). **e**, Enrichment (left) and number (right) of smoking-DMRs in CpG islands. **f**, Number of TFBSs enriched in hypermethylated and hypomethylated CpGs (smoking-DMPs) per chromatin state. **g**, Shared TFBSs enriched in hypomethylation across more than 8 chromatin states.

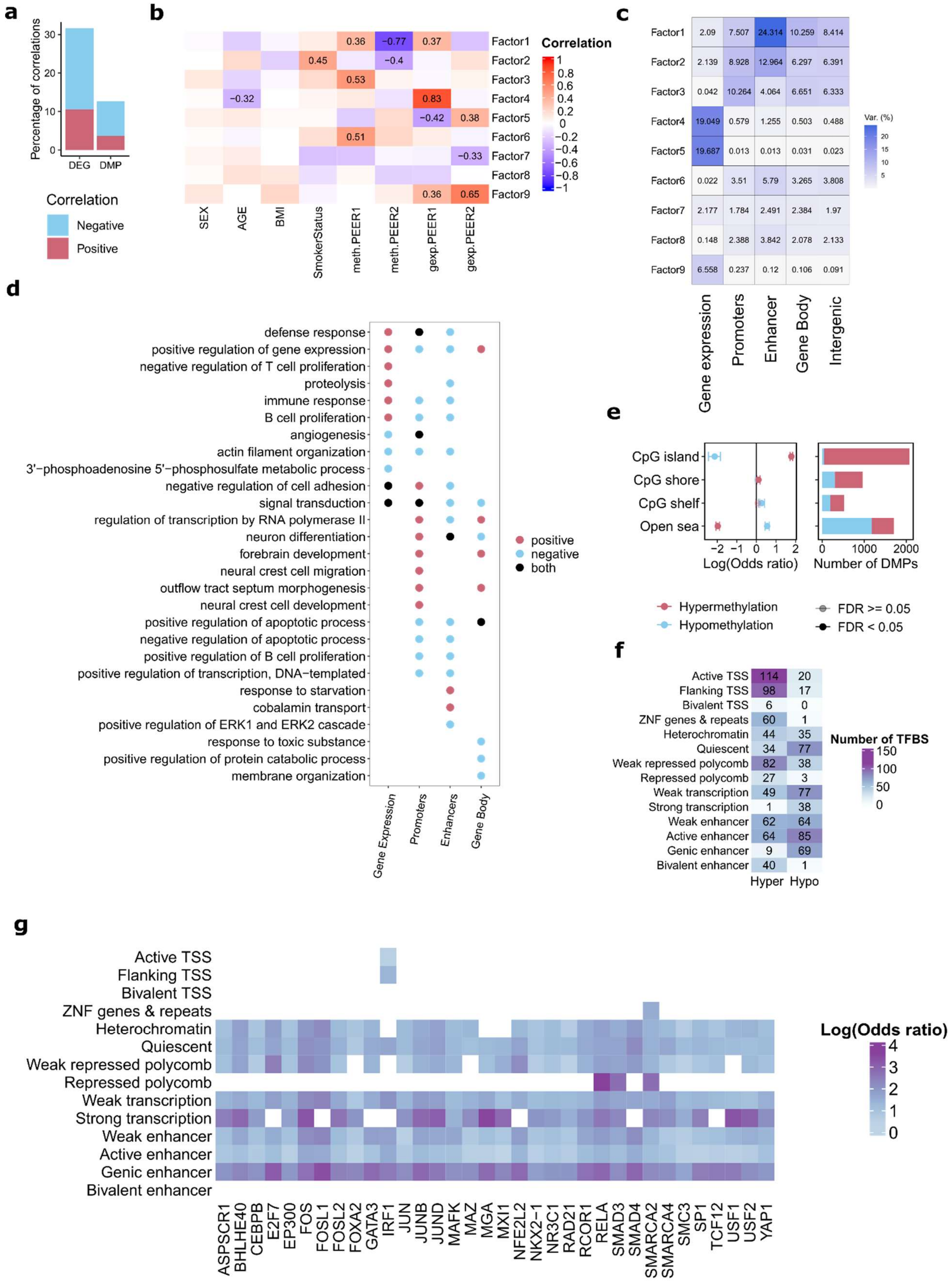
a
Percentage of correlations
30
20
10
0
DEG
DMP
Correlation
Negative
Positive
b
Factor1
Factor2
Factor3
Factor4
Factor5
Factor6
Factor7
Factor8
Factor9
0.36 −0.77 0.37
0.45 −0.4
0.53
−0.32 0.83
−0.42 0.38
0.51
−0.33
0.36 0.65
SEX
AGE
BMI
SmokerStatus
meth.PEER1
meth.PEER2
gexp.PEER1
gexp.PEER2
Correlation
1
0.8
0.6
0.4
0.2
0
−0.2
−0.4
−0.6
−0.8
−1
c
Factor1 2.09 7.507 24.314 10.259 8.414
Factor2 2.139 8.928 12.964 6.297 6.391
Factor3 0.042 10.264 4.064 6.651 6.333
Factor4 19.049 0.579 1.255 0.503 0.488
Factor5 19.687 0.013 0.013 0.031 0.023
Factor6 0.022 3.51 5.79 3.265 3.808
Factor7 2.177 1.784 2.491 2.384 1.97
Factor8 0.148 2.388 3.842 2.078 2.133
Factor9 6.558 0.237 0.12 0.106 0.091
Gene expression
Promoters
Enhancer
Gene Body
Intergenic
Var. (%)
20
15
10
5
d
defense response
positive regulation of gene expression
negative regulation of T cell proliferation
proteolysis
immune response
B cell proliferation
angiogenesis
actin filament organization
3'−phosphoadenosine 5'−phosphosulfate metabolic process
negative regulation of cell adhesion
signal transduction
regulation of transcription by RNA polymerase II
neuron differentiation
forebrain development
neural crest cell migration
outflow tract septum morphogenesis
neural crest cell development
positive regulation of apoptotic process
negative regulation of apoptotic process
positive regulation of B cell proliferation
positive regulation of transcription, DNA−templated
response to starvation
cobalamin transport
positive regulation of ERK1 and ERK2 cascade
response to toxic substance
positive regulation of protein catabolic process
membrane organization
Gene Expression
Promoters
Enhancers
Gene Body
positive
negative
both
e
CpG island
CpG shore
CpG shelf
Open sea
−2 −1 0 1 2
Log(Odds ratio)
0 1000 2000
Number of DMPs
Hypermethylation
Hypomethylation
FDR >= 0.05
FDR < 0.05
f
Active TSS 114 20
Flanking TSS 98 17
Bivalent TSS 6 0
ZNF genes & repeats 60 1
Heterochromatin 44 35
Quiescent 34 77
Weak repressed polycomb 82 38
Repressed polycomb 27 3
Weak transcription 49 77
Strong transcription 1 38
Weak enhancer 62 64
Active enhancer 64 85
Genic enhancer 9 69
Bivalent enhancer 40 1
Hyper
Hypo
Number of TFBS
150
100
50
0
g
Active TSS
Flanking TSS
Bivalent TSS
ZNF genes & repeats
Heterochromatin
Quiescent
Weak repressed polycomb
Repressed polycomb
Weak transcription
Strong transcription
Weak enhancer
Active enhancer
Genic enhancer
Bivalent enhancer
ASPSCR1
BHLHE40
CEBPB
E2F7
EP300
FOS
FOSL1
FOSL2
FOXA2
GATA3
IRF1
JUN
JUNB
JUND
MAFK
MAZ
MGA
MXI1
NFE2L2
NKX2−1
NR3C1
RAD21
RCOR1
RELA
SMAD3
SMAD4
SMARCA2
SMARCA4
SMC3
SP1
TCF12
USF1
USF2
YAP1
Log(Odds ratio)
4
3
2
1
0

Fig. S7 - Correlation between DNA methylation and expression and enrichments of smoking-age-DMPs. **a**, Percentage of DEG-DMP significantly correlated over the total number of DEGs or DMPs tested. **b,** Heatmap of correlation values (spearman) between inferred MOFA factors and demographic / technical variables. Only correlation values with FDR < 0.05 are represented. **c,** Variance explained by each factor across data modalities. **d,** Enrichment analysis of factor 2 across data modalities. Enrichment analysis was performed for positive and negatively correlated features independently. The results were summarised using *orsum* **e**, Enrichment of smoking-age-DMPs depending on CpG context. **f**, Number of TFBSs enriched in hypermethylated and hypomethylated CpGs per chromatin state for smoking-age-DMPs **g**, Shared TFBSs enriched in hypomethylation across more than 6 chromatin states for smoking-age-DMPs.

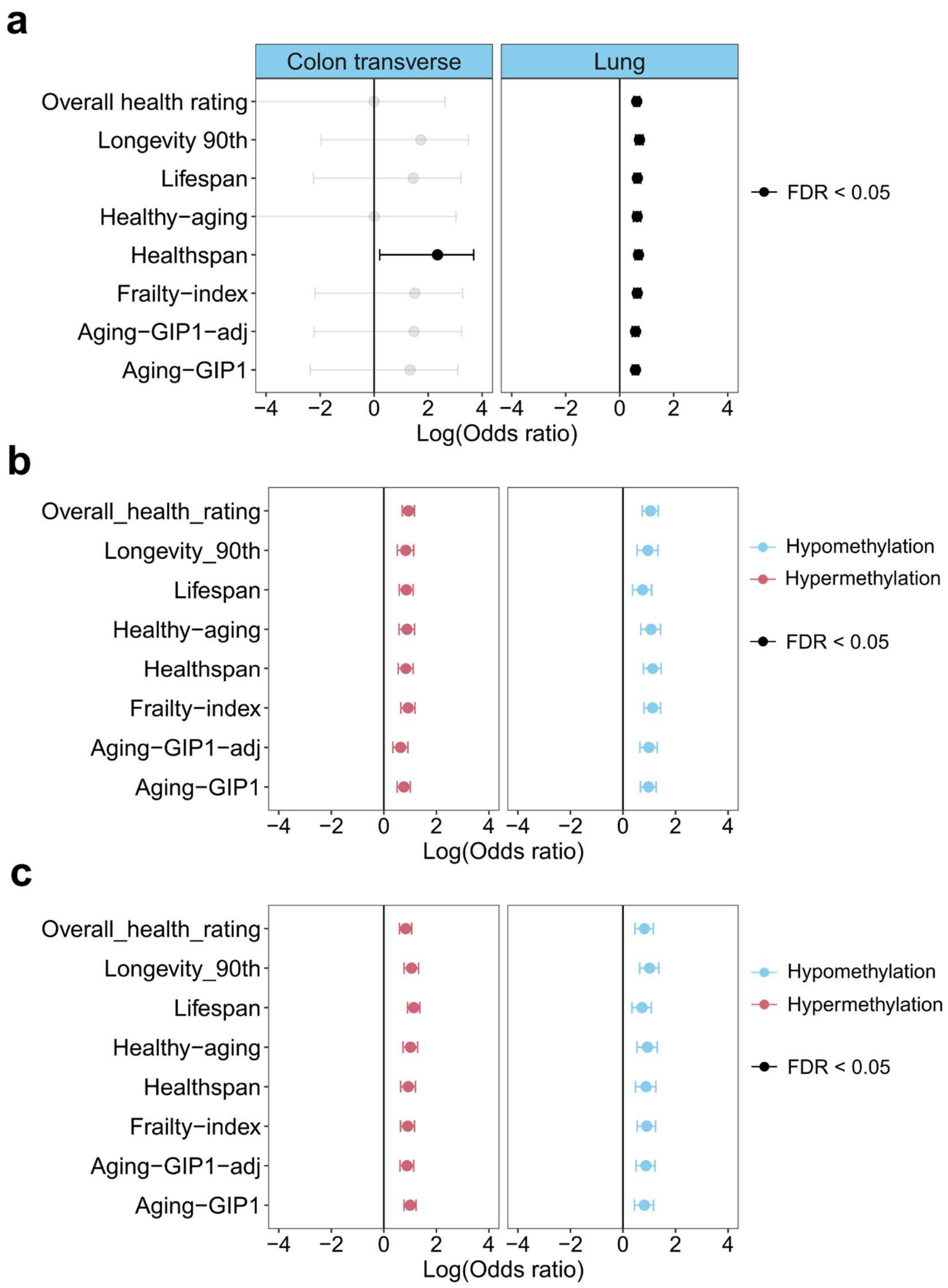

Fig. S8 - Overlap between smoking-DMPs and causal aging CpG. a, Overlap between smoking-DMP in colon transverse (left) and lung (right) with causal aging CpG position from Ying et al. (63). **b-c,** Overlap between smoking-DMPs in lung with damaging (**b**) and protective (**c**) CpG positions, discriminated by hypermethylated positions (in red) and hypomethylation positions (in blue).

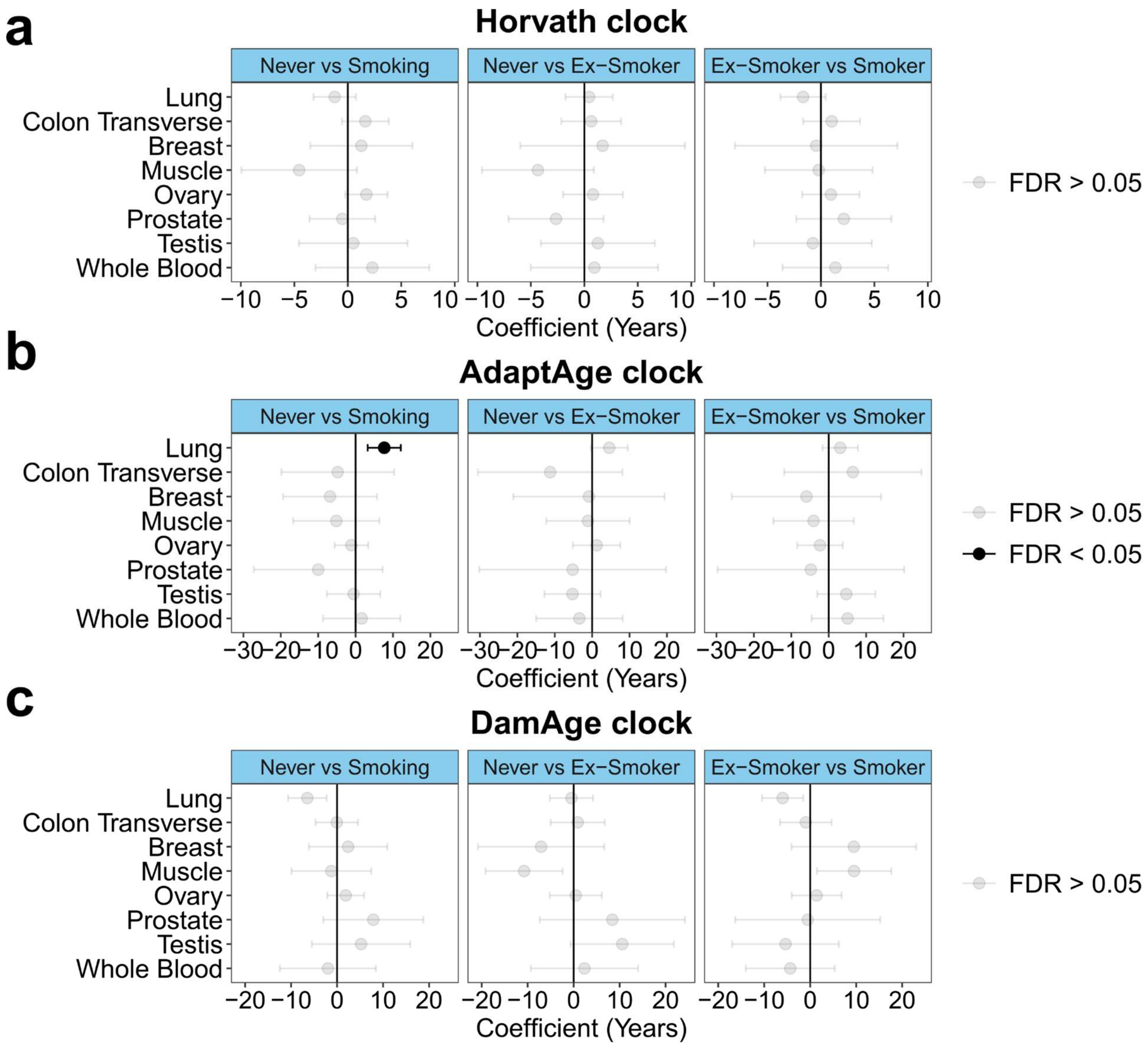

Fig. S9 - Methylation clock age in smokers, ex-smokers and never smokers across tissues. a-c, Coefficient of the linear regression on error values between never smokers and smokers, never smokers and ex-smokers, and ex-smokers and smokers for three molecular clocks: Horvath **(a)**, AdaptAge **(b)** and DamAge (**c**). We fitted a linear model for each tissue in each clock, adjusting for potential demographic and technical confounding factors (Methods). The error bars represent the 95% confidence intervals.

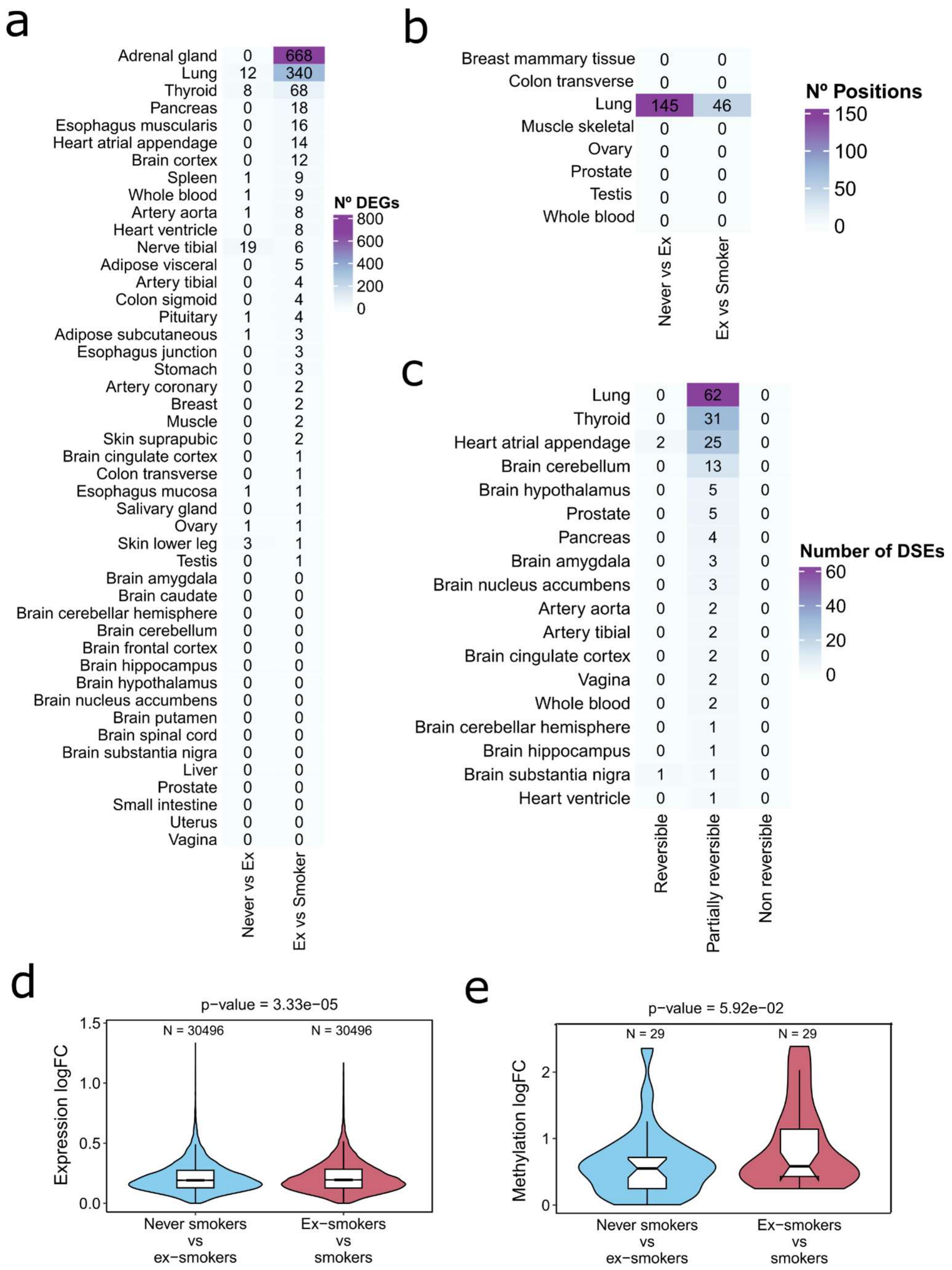

Fig. S10 - **Reversibility analysis**. **a**, Number of differentially expressed genes between never smokers and ex-smokers, and ex-smokers and smokers. **b**, Number of differentially expressed positions between never smokers and ex-smokers, and ex-smokers and smokers. **c**, Number of reversible and non reversible splicing events. **d,** smoking-DEGs and **e**, smoking-DMP log fold change in never vs ex-smokers and in ex-smokers vs smokers when analysis was constrained to the common samples between methylation and gene expression.

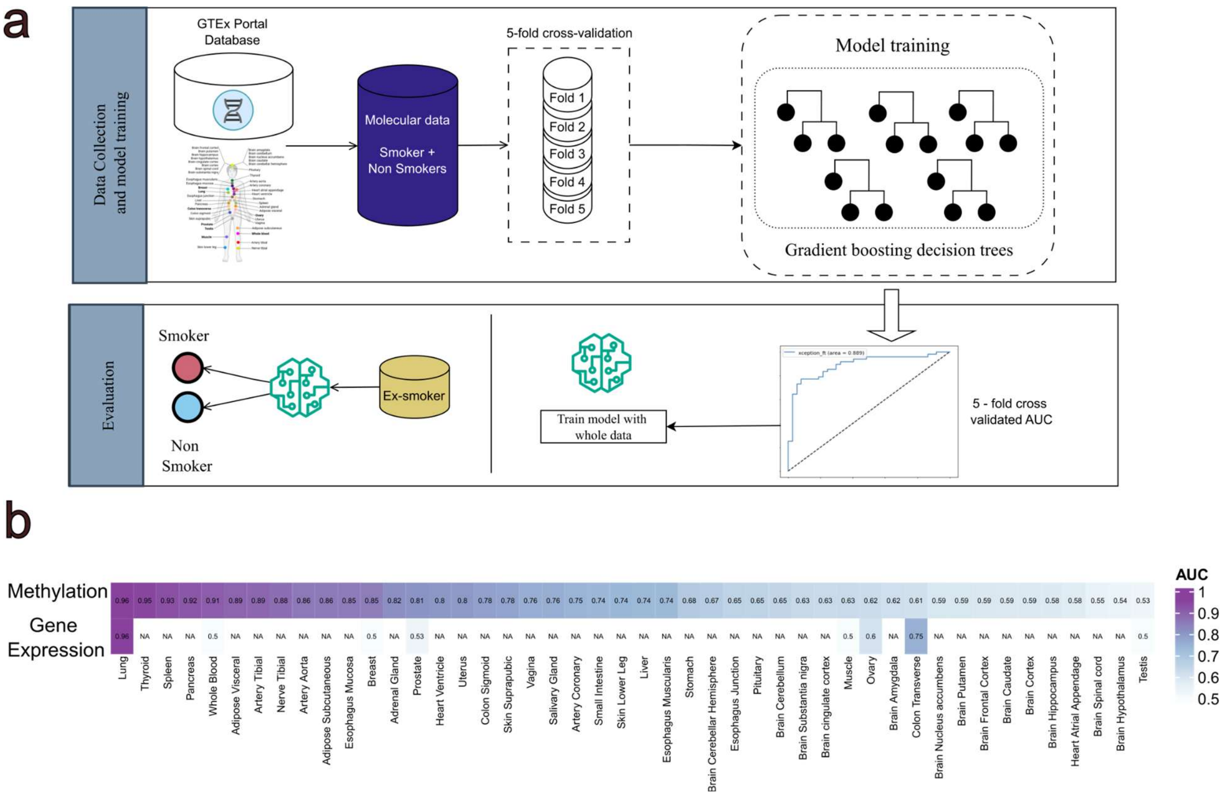

Fig. S11 - Machine learning to reclassify ex-smokers. **a**, General scheme of the machine learning approach used in this work for gene expression and methylation. Briefly, never smokers and smokers samples were considered as the target classes for model training. Performance was assessed using a 5-fold cross-validation scheme. After assessment, the binary classification model using all samples was trained to predict in which of these classes (smokers and never-smokers) the ex-smokers samples were classified. **b**, AUC across tissues (average across 5-fold cross-validation) for methylation and gene expression data. Only models with good accuracy (defined as AUC > 0.85) were considered for classification of ex-smokers samples.
